# Long-Term Fertilization Strategy Impacts *Rhizoctonia solani*–Microbe Interactions in Soil and Rhizosphere and Defense Responses in Lettuce

**DOI:** 10.3390/microorganisms10091717

**Published:** 2022-08-26

**Authors:** Loreen Sommermann, Doreen Babin, Jan Helge Behr, Soumitra Paul Chowdhury, Martin Sandmann, Saskia Windisch, Günter Neumann, Joseph Nesme, Søren J. Sørensen, Ingo Schellenberg, Michael Rothballer, Joerg Geistlinger, Kornelia Smalla, Rita Grosch

**Affiliations:** 1 Department of Agriculture, Ecotrophology and Landscape Development, Anhalt University of Applied Sciences, 06406 Bernburg, Germany; 2 Julius Kühn-Institute (JKI)—Federal Research Centre for Cultivated Plants, Institute for Epidemiology and Pathogen Diagnostics, 38104 Braunschweig, Germany; 3 Plant-Microbe Systems, Leibniz Institute of Vegetable and Ornamental Crops (IGZ), 14979 Großbeeren, Germany; 4 Institute of Network Biology, Helmholtz Zentrum München, German Research Center for Environmental Health, 85764 Neuherberg, Germany; 5 Institute of Crop Science (340h), University of Hohenheim, 70599 Stuttgart, Germany; 6 Section of Microbiology, Department of Biology, University of Copenhagen, 2100 Copenhagen, Denmark

**Keywords:** organic and mineral fertilization, *Rhizoctonia solani*, fungal ITS sequences, plant gene expression, 16S rRNA gene, high-throughput amplicon sequencing, *Talaromyces*

## Abstract

The long-term effects of agricultural management such as different fertilization strategies on soil microbiota and soil suppressiveness against plant pathogens are crucial. Therefore, the suppressiveness of soils differing in fertilization history was assessed using two *Rhizoctonia solani* isolates and their respective host plants (lettuce, sugar beet) in pot experiments. Further, the effects of fertilization history and the pathogen *R. solani* AG1-IB on the bulk soil, root-associated soil and rhizosphere microbiota of lettuce were analyzed based on amplicon sequencing of the 16S rRNA gene and ITS2 region. Organic fertilization history supported the spread of the soil-borne pathogens compared to long-term mineral fertilization. The fertilization strategy affected bacterial and fungal community composition in the root-associated soil and rhizosphere, respectively, but only the fungal community shifted in response to the inoculated pathogen. The potential plant-beneficial genus *Talaromyces* was enriched in the rhizosphere by organic fertilization and presence of the pathogen. Moreover, increased expression levels of defense-related genes in shoots of lettuce were observed in the soil with organic fertilization history, both in the absence and presence of the pathogen. This may reflect the enrichment of potential plant-beneficial microorganisms in the rhizosphere, but also pathogen infestation. However, enhanced defense responses resulted in retarded plant growth in the presence of *R. solani* (plant growth/defense tradeoff).

## 1. Introduction

Preservation of natural environments, including soil quality and fertility, is one of the major global challenges. Crop productivity, as certainly the main source of our food, depends on soil health. However, high input of synthetic agrochemicals in the long term exhibits negative effects on soil functioning and quality by changing physico-chemical as well as biological soil properties [1,2,3]. Therefore, agricultural/horticultural plant production systems should be regarded as vital living and biologically active ecosystems.

The occurrence of plant pathogens, insects and weeds is responsible for around 25% of yield losses in economically relevant crops [4] and the major reason for the increasing use of agrochemicals. The introduction of pesticides in plant production systems breaks the link between organic amendments and soil fertility, resulting in a decrease in soil organic matter over time [5]. This affects not only physico-chemical and biological soil properties, but is also relevant for overall soil health. Plant pathogens are an integral part of soil microbial communities, and a decline in soil health was shown to be accompanied by the accumulation of soil-borne pathogens in agroecosystems [6]. The maintenance of soil health—for instance, through balanced crop rotation, reduced tillage practices or application of organic fertilizers—is considered to be important for disease control [7,8]. Therefore, such environmentally friendly strategies in plant disease control should be further investigated to gain more relevance in agricultural practice as a sustainable alternative.

The ability of soils to suppress plant pathogens can be regarded as a manifestation of ecosystem stability and health, which is mediated to a large extent by soil microorganisms (general suppressiveness). Soil microbiota may control soil-borne pathogens through competition, antibiosis, parasitism or the improvement of plant immune responses [9]. Mechanisms by which soils inhibit the activity of plant pathogens are described for specific suppressiveness, which is only effective against one or a few pathogens [10,11]. Studies focusing on specific soil suppressiveness demonstrated that soil microbial communities respond to pathogen biomass accumulation, as found, e.g., for the “take-all” causing agent *Gaeumannomyces graminis* in wheat monoculture [12]. Hence, modification of the soil microbiota may contribute to plant protection through competitive effects or the enrichment of antagonists.

The understanding of the functions and interactions among soil microorganisms in agroecosystems is still limited. Several studies have highlighted how soil microbial communities are influenced by farming practices [13,14,15]. Many efforts have been made in understanding the essential relationships between soil and plant microbiota for soil functioning and plant performance [13,16,17,18,19,20,21]. This includes the beneficial effects of organic amendments on microbial diversity in the soil [13,22], linked with suppressive effects against soil-borne pathogens [9,23,24,25,26,27]. However, research on soil suppressiveness has not yet achieved solutions to manage soil-borne pathogens [28,29]. The knowledge of plant responses towards rhizosphere microbiota assemblages shaped by agricultural management strategies is limited, and only a few studies have addressed the long-term effects of mineral and organic fertilization on the soil microbiota and their suppressiveness against soil-borne pathogens [30,31,32,33].

A recent study with contrasting soil types from two long-term field experiments (LTEs), each with a long-term organic and mineral fertilization history, showed that soil legacies induced by fertilization strategies shaped the bacterial and archaeal communities in soil, as well as in the rhizosphere of the model plant, lettuce, independent of the soil origin [34]. The results highlighted that several genes involved in plant defense signaling were upregulated in lettuce when grown in soils under long-term organic compared to mineral fertilization, which indicated an induced plant physiological status. These so-called defense-priming beneficial microorganisms [35,36], such as members of Bacillales and other taxa, were enriched in the rhizosphere of lettuce grown in organic-fertilized soils [34]. In the present study, two model plant pathogen systems, lettuce *Rhizoctonia solani* AG1-IB and sugar beet *Rhizoctonia solani* AG2-2IIIb (teleomorph *Thanatephorus cucumeris*), were used to investigate soil suppressiveness depending on the fertilization strategy based on the spread of the pathogens in soil. For the analysis of plant responses in the presence of *R. solani* AG1-IB, a pot experiment with lettuce was performed. In addition, this work aimed to answer the question of whether the presence of the inoculated model pathogen *R. solani* AG1-IB in the soil alters the soil microbiota and consequently the assembly of the rhizosphere microbial communities and health of the host plant lettuce depending on the long-term fertilization strategy. It was hypothesized that both previously observed defense priming/induced systemic resistance (ISR) by beneficial microorganisms and suppression of plant pathogens by soil microbiota modulation in the rhizosphere of lettuce grown in organic-fertilized soil can contribute to the disease control of *Rhizoctonia*, as compared to plants grown in mineral-fertilized soil.

## 2. Materials and Methods

### 2.1. Field Site and Soil Sampling Strategy

The long-term field experiment of Humboldt University, Berlin (designated as HUB-LTE), located in Thyrow (Germany; 52°16′ N, 13°12′ E), was established in 2006. The soil was classified as Albic Luvisol [37]. This LTE provides access to soils with long-term organic (HU-org) and mineral (HU-min) fertilization practices. Soils were collected after the growing seasons in 2015, 2016 and 2017. In each year, 15 soil cores were randomly taken from the upper 30 cm soil horizons across the respective fertilization treatments and combined into a composite sample. Afterwards, soil samples were air-dried, sieved (4 mm mesh) and stored in the dark at 6 °C until use in growth chamber experiments. Soil characteristics, management practices and physiological parameters of the used soils are summarized in Windisch et al. [38].

### 2.2. Pathogens Used

The soil-borne pathogen *R. solani* AG1-IB (isolate 7/3/14, accession number AJ868459) causes bottom rot of lettuce (*Lactuca sativa* L.) and *R. solani* AG2-2IIIb (isolate BBA69670, accession numbers CYGV01000001–CYGV01002065) damping-off disease of sugar beet (*Beta vulgaris* L.). Both isolates were used in growth chamber bioassays for the assessment of soil suppressiveness in three consecutive years (2015, 2016, 2017). The impact of *R. solani* on plant health and rhizosphere microbiota was studied in the lettuce–*R. solani* AG1-IB plant pathogen system with soils from 2017 since lettuce is a well-established model plant for plant–microbial interaction studies [34]. The inocula of the *R. solani* isolates were prepared with barley kernels, which were sterilized before pathogen inoculation by autoclaving (121 °C for 30 min) three times with 24 h intervals, as described by Schneider et al. [39].

### 2.3. Assessment of Soil Suppressiveness

Disease spread of *R. solani* AG1-IB and *R. solani* AG2-2IIIb was determined after pathogen inoculation by scoring brown lesions or damping-off symptoms on the stems of lettuce and sugar beet seedlings at soil level using a similar method as described by Postma et al. [9]. The experiments were performed in a growth chamber (York, Mannheim, Germany; 20 °C/15 °C, 420 µmol m^−2^ s^−1^ photosynthetic active radiation, 60%/80% relative humidity, 16 h/8 h day/night). Pots (20 × 9.5 × 6 cm) were used with florist’s foam blocks at the bottom (Baumann Creative, Westhausen, Germany; water holding capacity approx. 55%). In each pot, 600 mL soil was filled on top of a water-saturated foam block. Lettuce (cv. Tizian, Syngenta, Bad Salzuflen, Germany) or sugar beet (cv. Lisanna, KWS Saat SE & Co. KGaA, Einbeck, Germany) were seeded in eight lines at 2 cm distance (0.5 cm and 2 cm deep, respectively; nine seeds per line). After germination, one infested barley kernel with the respective *R. solani* isolate was placed slightly beneath the soil surface in front of each lettuce or sugar beet seedling row. Disease spread was assessed weekly by counting the respective host plants exhibiting symptoms per row. Each treatment included three replicates arranged in a randomized design. This experiment was conducted three times with soils collected in three consecutive years (2015, 2016, 2017).

### 2.4. Growth Chamber Experiments to Study Lettuce Health and Rhizosphere Microbiota

To study the effect of the bottom rot pathogen *R. solani* AG1-IB on lettuce growth, health and rhizosphere microbiota, soils from the growing season 2017 were applied. Lettuce (cv. Tizian) was grown in the absence (HU-org, HU-min) and presence of the pathogen *R. solani* AG1-IB (HU-org + Rs, HU-min + Rs). The soils were initially incubated at the intended cultivation conditions for lettuce (20 °C/15 °C, 60%/80% relative humidity day/night) in the dark for 2 weeks. During the experiment, the water potential was regulated to 100 hPa (T5 tensiometer, UMS AG, Munich, Germany). Single lettuce plants were sown into pots (10 × 10 × 11 cm) filled with the respective soils and exposed to a temperature of 18 °C and 80% relative humidity for 2 days and afterwards further cultivated in a growth chamber at the above-mentioned conditions. To ensure the availability of comparable amounts of nitrogen (N) in each treatment, the N content of soils was analyzed in the beginning and adjusted to the recommendations for lettuce (0.32 g N per pot) using calcium nitrate in two portions (each 50%), before sowing and 3 weeks later. Soils were inoculated with the pathogen shortly before lettuce sowing. For the presence of *R. solani* AG1-IB, each pot was inoculated with 12 infested barley kernels, which were placed at distances of 2 cm from the seed and at 3 cm depth. In control pots, the same number of autoclaved non-infested barley kernels was used. In addition, each treatment included one pot per replicate without lettuce to assess the impact of the pathogen on microbial communities in bulk soil. Four plants for each of the four replicates per treatment were arranged in a randomized block design. After 10 weeks of cultivation, plants were harvested, shoot and root dry masses determined, and samples for microbial analyses (soil and rhizosphere) and plant gene expression (leaves) were collected.

### 2.5. Analysis of Plant Gene Expression

The expression level of genes was analyzed for lettuce after a cultivation time of 10 weeks. A total of 18 target genes was selected from the *Lactuca sativa*, L. cv. Tizian draft genome at NCBI [40] based on the comparison with functional genes of *Arabidopsis thaliana* using the “Arabidopsis Information Resource” (www.arabidopsis.org, accessed on 12 April 2019, [41]). All primer pairs and target genes used in this study were previously described [34] and are listed in Supplementary Table S1. The glyceraldehyde-3-dehydrogenase gene served as an endogenous control for qPCR normalization. Four leaves from two plants per replicate were snap-frozen in liquid nitrogen. The RNeasy Plant Mini Kit (QIAGEN GmbH, Hilden, Germany) was used to extract total RNA from 100 mg pulverized lettuce leaves. After RNA quantification by a NanoDrop spectrophotometer (Thermo Fisher Scientific, Waltham, MA, USA), cDNA was synthesized from 2 μg of total RNA with the High-Capacity cDNA Reverse Transcription Kit with RNase Inhibitor (Applied Biosystems, Foster City, CA, USA). The following qPCR was performed in three technical replicates using the same conditions as described previously [34]. Specific PCR products were confirmed by melting curve analysis and gel electrophoresis before relative quantification applying the 2^−ΔΔCt^ method [42]. Data were first normalized to the endogenous control and then logarithmically transformed to fold change differences. The standard error of the mean was calculated from the average of the technical triplicates. PERMANOVA (10,000 permutations) analysis was performed based on Bray–Curtis dissimilarities calculated from ΔCt values in R (version 3.6.1) using package *vegan* [43] and subjected to Principal Coordinates Analysis (PCoA).

### 2.6. Collection of Bulk Soils, Root-Associated Soils and Rhizosphere Samples and Total Community DNA Extraction

The complete root systems of two plants per replicate were combined and intensely shaken in order to obtain the loosely adhering soil (here defined as root-associated soil). The roots were then washed briefly in sterile tap water and the remaining adhering soil was defined as rhizosphere. Subsequently, 5 g of roots were transferred to Stomacher bags with saline (1:10) and treated by a Stomacher 400 Circulator (Seward Ltd., Worthing, UK), followed by centrifugation according to Schreiter et al. [44] in order to recover rhizosphere microbial cells. Aliquots of the habitats’ bulk soil, root-associated soil and rhizosphere pellets were stored at −20 °C until total microbial community (TC) DNA extraction. Subsequently, TC-DNA was extracted from bulk soil, root-associated soil (0.5 g fresh weight) and total rhizosphere pellets using the FastPrep-24 bead-beating system and FastDNA Spin Kit for Soil and subsequently purified with the GeneClean Spin Kit (both MP Biomedicals, Santa Ana, CA, USA). TC-DNA quality was checked by 0.8% agarose gel electrophoresis.

### 2.7. Microbial Community Analyses

Bacterial and archaeal community analysis focused on rhizosphere and bulk soil samples, similar to Babin et al. [45]. Briefly, the V3-V4 region of the 16S rRNA gene was amplified using the primer pair 341F and 806R [46,47], modified after [48] (Supplementary Table S2). In a second PCR, Illumina-specific sequencing adapters and sample identifiers were added, followed by amplicon purification and equimolar pooling, as previously described [45]. High-throughput amplicon sequencing of 16S rRNA genes was performed on an Illumina^®^ MiSeq^®^ platform (Illumina, San Diego, CA, USA) with MiSeq v2 kit (2 × 250 bp) in paired-end mode, according to the manufacturer’s instructions. Unassembled raw amplicon data are available at the NCBI Sequence Read Archive (SRA, https://www.ncbi.nlm.nih.gov/sra, accessed on 16 August 2022) under accession number PRJNA725140.

After de-multiplexing and trimming with cutadapt [49], the UPARSE pipeline [50] was applied for sequence merging, dereplication, removal of singletons and clustering of sequences into operational taxonomic units (OTUs, ≥97% sequence similarity). Representative OTUs were classified with the classify.seqs command (80% confidence) from mothur [51] using the RDP classifier [52] training set, version no. 18. Sequences unclassified at the domain level or of non-bacterial origin were discarded, as well as all 16S-OTUs with <10 reads over the whole data set, resulting in a total of 5957 final OTUs and, on average, 49,442 quality-filtered sequences per sample. Since archaeal reads were found only among the rare OTUs (<10 reads), they were not further considered. Thus, we will refer to “bacterial community” in the following.

The PCR conditions for fungal community analysis based on the internal transcribed spacer (ITS2) were conducted for all three habitats (bulk and root-associated soils, rhizosphere) according to Sommermann et al. [14]. In brief, amplification was conducted in three independent PCRs per sample at different annealing temperatures (54 °C, 56 °C, 58 °C), using the primer pair ITS86F/ITS4 [53,54] (Supplementary Table S2) with 28 cycles (35 s annealing time). Primers were equipped with the standard 8-nucleotide Illumina barcodes for sample identification (Supplementary Table S3). For each PCR, 10 ng template DNA and bovine serum albumin (BSA; final concentration 0.5 mg ml^−^^1^) were added. Independent PCRs per sample were pooled and purified using the MinElute PCR Purification Kit (QIAGEN, Hilden, Germany) with a final elution step in 12 μL 10 mM Tris-HCl, pH 8.5. Subsequently, the concentration of each sample was determined by a Qubit^®^ fluorometer (Invitrogen, Carlsbad, CA, USA), followed by pooling of the amplicons to equimolar amounts. High-throughput sequencing of the ITS2 pool and the following taxonomic classification was processed as previously described [45] on the Illumina^®^ MiSeq^®^ platform using the MiSeq v3 kit (2 × 300 bp) in paired-end mode. Unassembled raw sequences were submitted to the European Nucleotide Archive (ENA) under the following BioProject accession number: PRJEB53229. Barcode primer and adapter trimming was performed including the FASTX toolkit [55] and then merged using FLASH v.1.2.10 [56] with a minimum overlap of 10 bp. A database-dependent strategy according to Antweiler et al. [57] with a local GALAXY Bioinformatics Platform in combination with the fungal UNITE database v8.0 [58,59] was conducted with the sequences by applying a closed reference approach. In brief, all sample sequences were aligned with the database (e-value ≤ 0.001) and only results with minimum alignment length ≥200 bp and similarity ≥97% were kept. In summary, 2,940,507 out of 4,252,478 sequences (69.5% ± 11.5% of all 48 samples) remained. The SH numbers of the UNITE database were used as identifiers for the ITS-OTU abundance table generated by counting the sequences per taxonomic assignment. Finally, a total of 1227 OTUs was obtained, with an average of 61,260 reads per sample.

A qPCR approach according to Wallon et al. [60] was conducted to quantify the abundance of the inoculated pathogen *R. solani* AG1-IB in soils of the growth chamber experiment. Amplification was performed in a final volume of 20 µL containing PowerUp^TM^ SYBR^TM^ Green Master Mix (Applied Biosystems, Vilnius, Lithuania), 0.5 µM of each primer (AG1-IB-F3, AG1-IB-R [60]; Supplementary Table S2), 0.5 mg mL^−1^ BSA and 10 ng template DNA. The qPCR of four biological replicates was performed with the QuantStudio 5 qPCR System (Applied Biosystems, Darmstadt, Germany), each in four technical replicates. The thermal program consisted of initial heating (50 °C for 2 min) and denaturation (95 °C for 10 min) followed by 40 cycles: 95 °C for 5 s, 62 °C for 20 s. For quantification, a standard curve of serially diluted *R. solani* AG1-IB DNA was generated under the same conditions in five technical replicates (R^2^ = 0.999, efficiency = 94.1%).

### 2.8. Statistical Analysis

A linear mixed model was used to predict the effects of fertilization strategy and *R. solani* AG1-IB inoculation on the shoot and root dry masses of lettuce. The model included replicates as a random effect. Tukey’s HSD tests were performed post-hoc and heteroscedasticity was accounted for by using group variances. The spread (*v_d_*) (cm day^−1^) of the *R. solani* pathogens was analyzed using the procedure ROBUSTREG in SAS 9.4 (SAS Institute Inc. 2019). The estimated slope *a* and its 95% confidence limits (CLs) of the model *r*×*d* = a×*t* + b, where *r* represents the last row of plants showing symptoms, *d* is the distance between rows (cm) and *t* is the number of days since inoculation, were used as estimates for *v_d_* and its CLs, respectively. Comparison of treatments was performed by observation of overlapping or non-overlapping CLs. 

Multivariate analyses of microbial communities were carried out in R [61] using the following packages: *vegan* [43], *pheatmap* [62], *car* [63], *rcompanion* [64], *agricolae* [65], *plyr* [66], *edgeR* [67,68], *phyloseq* [69], *ggplot2* [70], *RColorBrewer* [71], *MASS* [72] and *mvabund* [73]. Alpha diversity indices (species richness, Shannon index) were determined based on subsampling to the lowest number of reads (17,505 for 16S rRNA gene or 31,967 for ITS2 data set, respectively). The effects of the fertilization strategy and the presence of *R. solani* AG1-IB on the fungal communities were tested by PERMANOVA (10,000 permutations) based on a Bray–Curtis dissimilarity matrix of count data. For 16S rRNA gene count data, a generalized linear model under negative binomial distribution was used, followed by analysis of deviance to test for the effect of factors “fertilization” and “*R. solani* presence” (likelihood ratio test, 999 bootstrap iterations). Non-metric multidimensional scaling (NMDS) analyses were conducted based on Bray–Curtis dissimilarities calculated from count data for both bacterial and fungal data sets. The mean relative abundance of the 30 most abundant microbial genera in each treatment was visualized by heatmaps (Euclidean distance clustering). Fertilization-dependent (HU-org vs. HU-min) or inoculation-dependent (absence vs. presence of *R. solani*) relative abundances of bacterial and fungal genera were analyzed by likelihood ratio tests under negative binomial distribution and generalized linear models (edgeR) separately per habitat, considering interaction effects between factors. The effects of fertilization and pathogen inoculation on microbial alpha diversity indices, relative abundances of phyla and qPCR abundance of the inoculated *R. solani* AG1-IB were tested separately for each habitat by using two-way analysis of variance (ANOVA) followed by post-hoc Tukey’s HSD test (*p* < 0.05). Data transformation by Tukey’s Ladder of Powers was carried out, if ANOVA assumptions failed. The qPCR abundance data of *R. solani* per gram bulk soil had to be transformed into ranks to obtain valid results for normal distribution and variance homogeneity.

## 3. Results

### 3.1. Long-Term Mineral Fertilization Reduced the Spread of Rhizoctonia solani

Soil suppressiveness was analyzed by measuring the rate of disease spread of *R. solani* AG1-IB on lettuce seedlings and of *R. solani* AG2-2IIIb on sugar beet seedlings in six independent experiments with soil samples from the growing seasons in 2015, 2016 and 2017. By assessing the effect of the long-term fertilization, a significantly lower spread of both *R. solani* pathogens was observed in mineral- compared to organic-fertilized soils in each sampling year, except for AG2-2IIIb in the soil sampled in 2017 (Table 1). In addition, no significant differences in hyphal spread were determined depending on the sampling year (2015: 0.48 ± 0.09 cm day^−1^, 2016: 0.40 ± 0.16 cm day^−1^, 2017: 0.49 ± 0.09 cm day^−1^), without considering isolates or long-term fertilization history. However, averaged over all three years, a significantly faster spread was revealed for the pathogen *R. solani* AG2-2IIIb (0.59 ± 0.19 cm day^−1^) compared to *R. solani* AG1-IB (0.32 ± 0.09 cm day^−1^).

### 3.2. Fertilization Strategy and Presence of R. solani AG1-IB Limited Lettuce Growth

**Figure 1:**
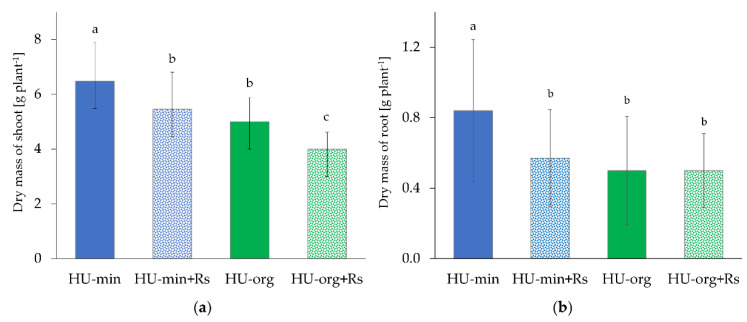
Influence of long-term mineral (HU-min) and organic (HU-org) fertilization and presence of the pathogen *Rhizoctonia solani* AG1-IB (+Rs) on shoot (**a**) and root (**b**) dry masses of lettuce (cv. Tizian) grown under controlled growth chamber conditions. Different letters indicate significant differences according to linear mixed model analysis (*p* < 0.05).

Lettuce plants were cultivated for 10 weeks in a growth chamber in soils (from 2017) under a long-term mineral or organic fertilization strategy. Significant effects of the factors long-term fertilization (*p* < 0.001) and *R. solani* AG1-IB inoculation (*p* < 0.05) on lettuce growth were revealed based on the linear mixed model. Significantly lower shoot (23%) and root (40%) dry masses of lettuce were detected in soil under long-term organic fertilization compared to the mineral-fertilized soil (HU-min vs. HU-org; Figure 1a,b).

The pathogen *R. solani* AG1-IB reduced the shoot growth of lettuce (16% in HU-min + Rs vs. HU-min; 20% in HU-org + Rs vs. HU-org) significantly, independently of fertilization strategy. The root dry mass was also significantly reduced (32%) by the pathogen in mineral-fertilized soil but not in the organic-fertilized soil (Figure 1b).

At the end of the cultivation period, the shoot nutritional status was analyzed and moderate deficiencies in nutrient concentrations such as N, P, K and S were identified in all treatments (Supplementary Table S4). Only for K, significant differences related to the fertilization strategy were observed, showing higher values in the mineral treatment (HU-min, HU-min + Rs). Other macro- and micronutrients in the shoot tissues, such as Ca, Mg, Mn and Zn, reached the sufficiency range in all treatments.

### 3.3. R. solani AG1-IB and Fertilization Strategy Influenced Gene Expression Profiles of Lettuce

To determine whether gene expression levels in lettuce were influenced by the different long-term fertilization strategies and the presence of the pathogen *R. solani* AG1-IB, qPCR for 18 different plant genes was performed (Supplementary Figure S1). PERMANOVA analyses confirmed that the fertilization strategy (HU-org/HU-min) had a moderate influence (explained variance 11.6%), while no significant effects of the pathogen on gene expression levels were found (Table 2). The interaction of the factors fertilization strategy and pathogen presence had the highest influence (explained variance 43.7%) on gene expression patterns. When calculated separately for each fertilization strategy, the presence of the *R. solani* AG1-IB explained 53.5% of variance in gene expression in lettuce when grown in mineral-fertilized soil and 60.5% when grown in organic-fertilized soil (both *p* < 0.05).

**Figure 2:**
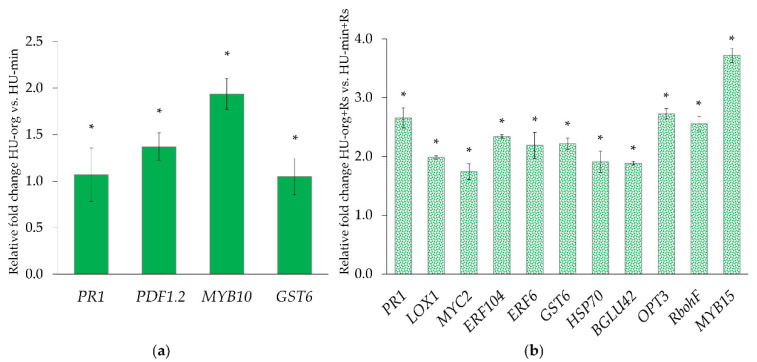
Relative fold changes of gene expression associated with biotic/abiotic stress signaling in lettuce (cv. Tizian): (**a**) plants grown in soils with long-term organic (HU-org) compared to mineral (HU-min) fertilization, (**b**) plants grown in presence of *Rhizoctonia solani* AG1-IB (+Rs) in organic- (HU-org + Rs) compared to plants grown in mineral-fertilized soil (HU-min + Rs). * Indicates significant differences according to Tukey’s HSD test (*p* < 0.05).

In the treatments without *R. solani* AG1-IB (HU-org vs. HU-min), the expression levels of *PR1*, *PDF1.2*, *MYB10* and *GST6* genes in lettuce shoots significantly increased when grown in organic-fertilized soil compared to plants grown in mineral-fertilized soil (Figure 2a). For all other analyzed genes, a lower level of expression in plants from organic- compared to mineral-fertilized soils was observed. However, the differences were not statistically significant.

In the presence of the pathogen *R. solani* AG1-IB, the plants from soils with long-term organic fertilization showed significantly increased expression of several genes involved in abiotic and biotic stress signaling (*PR1*, *LOX1*, *MYC2*, *ERF104*, *ERF6*, *GST6*, *HSP70*, *BGlu42*, *OPT3*, *RbohF* and *MYB15*) in comparison to lettuce grown in soil with mineral fertilization (HU-org + Rs vs. HU-min + Rs; Figure 2b).

### 3.4. Microbial Communities in Soil and Lettuce Rhizosphere

#### 3.4.1. Fertilization Strategy but Not *R. solani* AG1-IB Shifted Bacterial Community Composition

**Figure 3:**
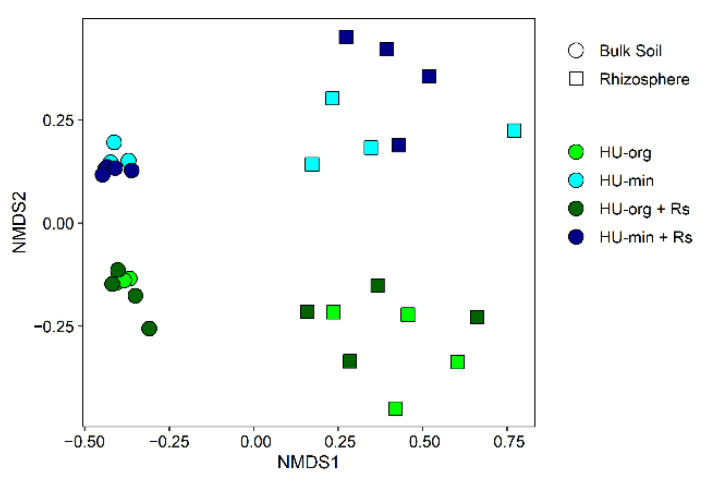
Non-metric multidimensional scaling (NMDS) of bacterial communities in bulk soil and rhizosphere (stress = 0.10) of lettuce (cv. Tizian) depending on long-term fertilization strategy (HU-org—organic fertilization; HU-min—mineral fertilization) and the presence of *Rhizoctonia solani* AG1-IB (+Rs).

Bacterial communities in the bulk soil and rhizosphere differed strongly (Figure 3). Fertilization strategy was the main driver of the bacterial community composition in bulk soil (deviance = 25,004 ***) and in the lettuce rhizosphere (deviance = 14,432 **), resulting in discrete clusters of samples with organic or mineral fertilization history in NMDS analysis (Figure 3). No significant effect of the pathogen *R. solani* AG1-IB on the total bacterial community was detected in both habitats (analysis of deviance; Figure 3).

**Figure 4:**
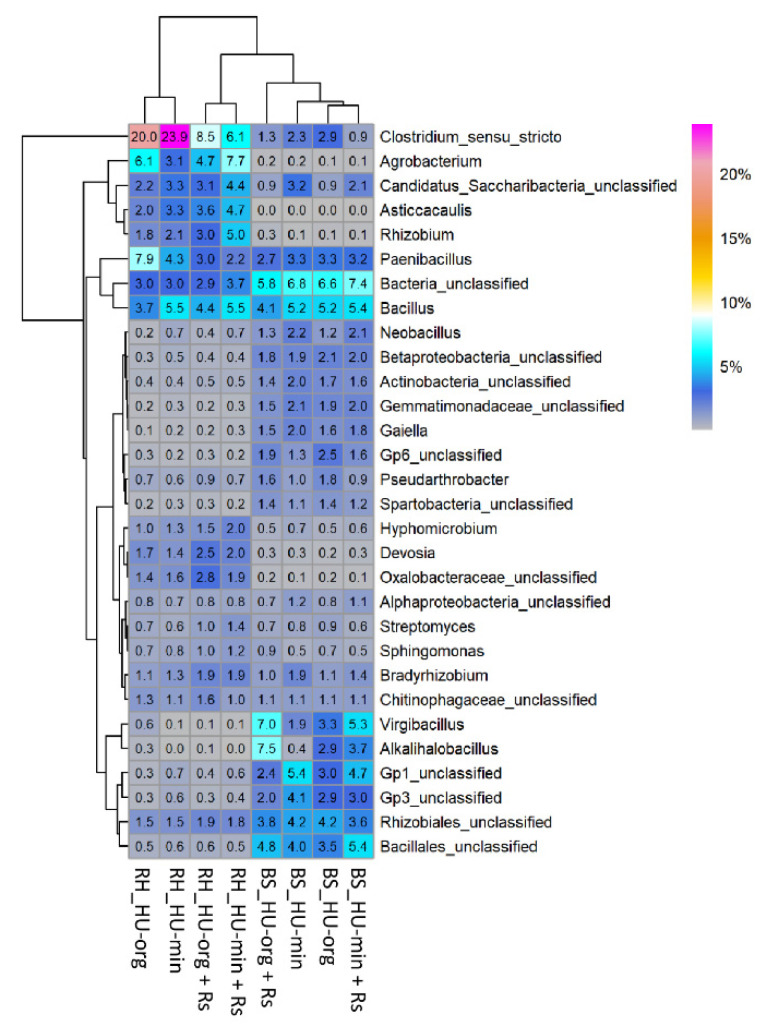
Relative abundance of the 30 major bacterial genera in bulk soil (BS) and rhizosphere (RH) of lettuce (cv. Tizian) grown in soils with organic (HU-org) or mineral (HU-min) fertilization history in the absence and in the presence of *Rhizoctonia solani* AG1-IB (+Rs).

Bacterial alpha diversity (species richness, Shannon index) was neither affected by long-term fertilization nor by pathogen inoculation (Supplementary Table S5). Only a few significant differences in the relative abundances of bacterial phyla due to fertilization (only in bulk soil) or pathogen presence (only in rhizosphere) were observed (Supplementary Table S6). In bulk soil, the candidate phylum Saccharibacteria exhibited a higher relative abundance (1–3%) in mineral- than in organic-fertilized soils. In the rhizosphere of lettuce, a significant enrichment of Gammaproteobacteria (5–9%) was observed in organic-fertilized soil in the presence of *R. solani* AG1-IB (HU-org + Rs; Supplementary Table S6). When looking at the 30 most dominant bacterial genera (Figure 4), it became apparent that different taxa predominated in the bulk soil (e.g., *Virgibacillus*, *Pseudarthrobacter*, acidobacterial groups Gp1, Gp3, Gp6) and rhizosphere (e.g., *Clostridium*, *Agrobacterium*, *Rhizobium*, *Asticcacaulis*, *Devosia*). In bulk soil, fertilization-dependent differences in relative abundance were revealed among bacterial taxa. For instance, acidobacteria Gp1 (Figure 4), *Tumebacillus* and sequences with the closest affiliation to Ktedonobacterales were significantly higher in soils under mineral fertilization, independent of pathogen presence (Table 3). Bulk soils with organic fertilization history (HU-org, HU-org + Rs) exhibited a higher relative abundance of acidobacteria Gp4 than mineral-fertilized soils (HU-min and HU-min + Rs). However, pathogen presence-dependent responders to fertilization were also identified in bulk soil (Table 3A,B). In response to *R. solani* AG1-IB, a significantly lower relative abundance of *Actinoallomurus* (0.1 ± 0%) or *Fontibacillus* (0 ± 0%) was observed in bulk soils with mineral or organic fertilization history, respectively, when compared to the controls without pathogen inoculation (0.7 ± 1.3%; 0.5 ± 0.7%, respectively). Moreover, only a few minor pathogen presence-dependent differences (FDR < 0.05; abundance < 0.5%) were observed in bulk soils (data not shown).

The comparison of the relative abundances of bacterial genera in the rhizosphere of lettuce grown in soils with different fertilization history or in the presence of *R. solani* AG1-IB showed statistically significant differences only among minor abundant genera (mean <0.5%; FDR < 0.05; data not shown). Notably, sequences identified only at higher taxonomic levels, e.g., Selenomonadales, exhibited a significantly higher relative abundance in the rhizosphere of mineral-fertilized soil (HU-min; 3 ± 3%) when compared to the organic treatment in the absence of *R. solani* (HU-org; 0.2 ± 0.2%) and to the mineral treatment in the presence of *R. solani* (HU-min + Rs; 0 ± 0%).

#### 3.4.2. Fertilization Strategy and *R. solani* AG1-IB Shaped Fungal Community Composition

**Figure 5:**
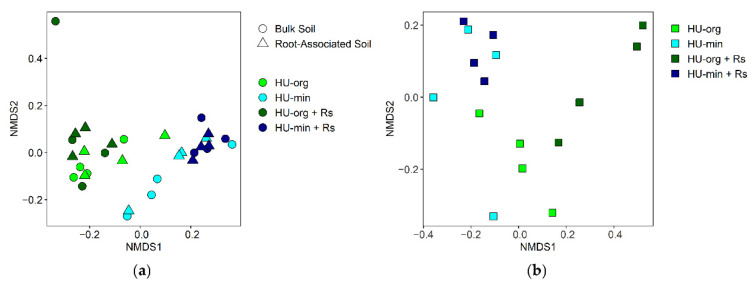
Non-metric multidimensional scaling (NMDS) of fungal communities (**a**) in bulk soil and root-associated soil (stress = 0.09), and (**b**) in the rhizosphere (stress = 0.11) of lettuce (cv. Tizian) depending on long-term fertilization strategy (HU-org—organic fertilization; HU-min—mineral fertilization) and the presence of *Rhizoctonia solani* AG1-IB (+Rs).

In each habitat (bulk soil, root-associated soil, rhizosphere), the effect of fertilization practice was the main driver of fungal community structures, especially in the root-associated soil (Table 4). NMDS ordination of the fungal communities showed a clear separation between organic and mineral fertilization in all habitats (Figure 5). Furthermore, fungal communities were significantly influenced by *R. solani* AG1-IB inoculation in the bulk soil and in the rhizosphere, but not in the root-associated soil (Table 4). Higher heterogeneity was observed among fungal communities in the rhizosphere of lettuce grown in organic-fertilized soil compared to mineral fertilization. Additionally, a distinct separation of fungal communities in the rhizosphere in organic soils depending on the presence/absence of the pathogen (HU-org, HU-org + Rs) was found (Figure 5b).

Alpha diversity indices (species richness, Shannon index) were calculated to assess the effects of the studied factors on fungal communities in each habitat. A significant influence of fertilization strategy on the fungal diversity was found in root-associated soil, resulting in higher diversity indices in organic vs. mineral treatments (Supplementary Table S7). Alpha diversity in bulk soil and the rhizosphere of organic treatments (HU-org, HU-org + Rs) was also higher as compared to mineral treatments (HU-min, HU-min + Rs) but not significant. The presence of *R. solani* AG1-IB reduced fungal diversity in all treatments except in root-associated soil with organic fertilization history, but the differences were not significant (Supplementary Table S7B).

The fertilization strategy affected the relative abundance of various phyla in all three habitats, whereas *R. solani* influenced only single phyla, such as Mortierellomycota in bulk soil and Chytridiomycota in the rhizosphere (Supplementary Table S8). The relative abundances of Ascomycota (HU-org vs. HU-min; 54–88% vs. 25–43%), Chytridiomycota (HU-org vs. HU-min; 0.1–1.5% vs. 0.1–0.3%), Glomeromycota (HU-org vs. HU-min, 0.1–1.7% vs. 0–0.3%) and Mortierellomycota (in bulk soil and root-associated soil, HU-org vs. HU-min; 12–16% vs. 6–11%) were enriched in organic-fertilized soils compared to mineral-fertilized soils. A higher relative abundance of Mucoromycota (HU-min vs. HU-org; 32–66% vs. 0.7–19%) was found in the mineral-fertilized soils of all habitats. In bulk and root-associated soils, the relative abundance of Mortierellomycota decreased in the presence of *R. solani* AG1-IB (Supplementary Table S8). The relative abundance of the Basidiomycota (phylum that R. *solani* belongs to) was not affected by any of the factors (Supplementary Table S8).

#### 3.4.3. *Rhizoctonia solani* AG1-IB Affected Relative Abundance of Fungal Taxa in Organic-Fertilized Soils

**Figure 6:**
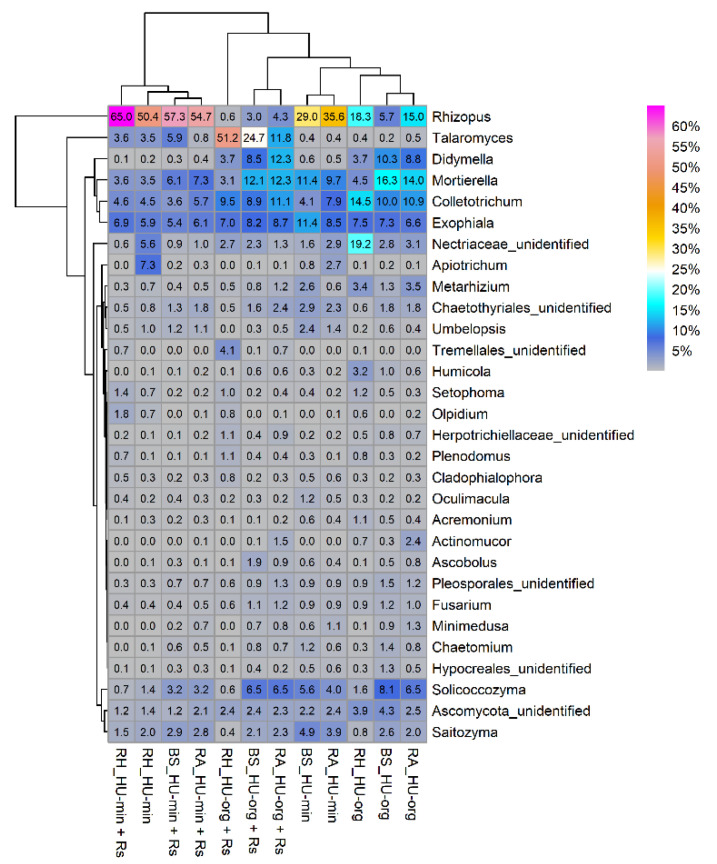
Relative abundance of the 30 most prevalent fungal genera in bulk soil (BS), root-associated soil (RA) and rhizosphere (RH) of lettuce (cv. Tizian) grown in soils with organic (HU-org) or mineral (HU-min) fertilization in the absence and the presence of *Rhizoctonia solani* AG1-IB (+Rs).

The fertilization strategy and the pathogen *R. solani* AG1-IB altered the relative abundances of the most predominant genera (Figure 6). A higher impact than that of *R. solani* was given by fertilization, resulting in prevalence differences in fungal genera between organic and mineral fertilization (Table 5 and Table 6). A high relative abundance of the genus *Rhizopus* was observed in all habitats of HU-min, especially in the presence of *R. solani* (HU-min vs. HU-org; HU-min + Rs vs. HU-org + Rs; Figure 6, Table 5). In the presence of *R. solani* in organic-fertilized soils, this genus exhibited a lower relative abundance in the rhizosphere of lettuce compared to *R. solani* absence (HU-org vs. HU-org + Rs; Figure 6, Table 6A).

In contrast, the relative abundance of *Talaromyces* increased significantly in all habitats of the organic-fertilized soils with *R. solani* (HU-org + Rs vs. HU-org; Figure 6, Table 6A) and was also enriched in bulk soils with mineral fertilization in the presence of the pathogen (HU-min + Rs vs. HU-min; Table 6B). A higher relative abundance of *Talaromyces* in the rhizosphere of lettuce was also found when grown in mineral- compared to organic-fertilized soil (HU-min vs. HU-org; Table 5A).

Several genera showed fertilization-dependent alterations in relative abundance. The genus *Didymella* was more prevalent in all habitats of organic-fertilized soils compared to mineral-fertilized soils, irrespective of the pathogen (Table 5, Figure 6). *Humicola* was more prevalent in organic-fertilized soils in all habitats in the absence of *R. solani* (Table 5A), but decreased similarly to *Arthrobotrys* in the rhizosphere in the presence of *R. solani* (Table 6A). With respect to sequence reads only identified at higher taxonomic levels, *Sordariales* were found to be enriched in bulk and root-associated soils of the organic treatments in the absence of *R. solani* AG1-IB (Table 5A). The genus *Funneliformis* (Glomeromycota) was also more prevalent in the rhizosphere of the organic treatments in the absence of *R. solani* (Table 5A), whereas the genera *Ilyonectria* and *Rhizophagus* were more prevalent in the rhizosphere of the organic treatments, independent of the presence of *R. solani* (Table 5A,B). *Umbelopsis* was enriched in all habitats of mineral-fertilized soils, independent of the presence of *R. solani* (Table 5A,B). The genera *Apiotrichum* and *Fusicolla* and sequences with the highest affiliation to the higher taxonomic level *Bionectriaceae* were enriched in root-associated soils and in the rhizosphere of mineral-fertilized soils in the absence of *R. solani* (Table 5A), but their relative abundances decreased in the rhizosphere of lettuce in the presence of *R. solani* (Table 6B).

#### 3.4.4. No Clear Indication of *R. solani* AG1-IB Establishment in the Differently Fertilized Soils

The specific amplification of pure *R. solani* AG1-IB DNA by qPCR was performed under similar PCR conditions as ITS2 amplicon generation using universal primers. Both approaches yielded clearly positive results in conventional PCR. For quantification of *R. solani* AG1-IB by qPCR, the standard curve was based on seven dilution levels (10–1.0 × 10^−5^ ng), but the lowest level could not be determined (below detection limit). *R. solani* was detected by qPCR only in three samples (one of each in root-associated soil of HU-min + Rs and in rhizosphere of HU-org + Rs and HU-min + Rs, respectively) within the calibration range. The remaining samples exhibited lower abundances outside the calibration range and were thus based on extrapolation. *Rhizoctonia solani* AG1-IB had a significantly higher abundance in the rhizosphere of inoculated soils compared to the absence of the pathogen (Table 7). A similar trend was observed in bulk soils. *R. solani* could not reliably be quantified in root-associated soils (close to/below detection limit). In addition, a significant effect of the fertilization strategy on *R. solani* AG1-IB abundance was observed in bulk soils, resulting in higher abundances in mineral fertilization. A similar trend was observed in the rhizosphere. 

Additionally, the presence of *R. solani* in the habitats was estimated by ITS amplicon sequencing based on four OTUs with the highest affiliation to *Thanatephorus cucumeris* (teleomorph of *R. solani*). Only in the bulk soil under organic fertilization and the presence of *R. solani*, *Thanatephorus* was detectable, but in low relative abundances (Supplementary Table S9). Furthermore, this genus showed no significant alterations depending on fertilization or presence/absence of *R. solani*. In contrast, the related genus *Waitea,* represented by one OTU with the highest affiliation to *W. circinata* (teleomorph of *Rhizoctonia zeae*), showed significantly higher relative abundances in the presence of *R. solani* AG1-IB in organic-fertilized bulk soils (HU-org + Rs vs. HU-org; Table 6A), however, depending on fertilization (HU-org + Rs vs. HU-min + Rs, Table 5B). Additionally, the relative abundances of *W. circinata* were significantly higher in the presence of *R. solani* in root-associated soils and in the rhizosphere of soils with organic fertilization (HU-org + Rs vs. HU-min + Rs, Supplementary Table S9B), as well as in the absence of *R. solani* in root-associated soils (HU-org vs. HU-min, Supplementary Table S9A).

## 4. Discussion

### 4.1. No Inhibition of the Spread of Rhizoctonia Pathogens in Organic-Fertilized Soil

Organic fertilization was reported to alter soil microbial communities and to enhance their diversity [13] and thus decrease the incidence of plant diseases caused by soil-borne pathogens [11]. It was hypothesized that these microbial factors play a key role in inhibition of pathogens such as *R. solani*. Contrary to our hypothesis, a consistently higher suppressiveness of mineral-fertilized soil (HU-min) against both *R. solani* model pathogens was found. Bonanomi et al. [26] reported that disease suppressiveness varied largely under organic fertilization depending on the pathogen and was effective against *R. solani* only in 26% of studied cases. Based on our data, we suggest that organic fertilizers provide saprotrophic pathogens such as *R. solani* with substrates and support their growth and spread [74]. Genome analysis of *R. solani* AG1-IB indicated its ability to feed on organic substrates and to produce toxic compounds [75], which may explain their high competitiveness. Furthermore, the lower spread of *R. solani* in the soil with mineral fertilization history may be related to long-term pesticide use.

### 4.2. Long-Term Organic Fertilization Impacted R. solani AG1-IB Interaction with Indigenous Soil Fungi

Fungal communities are critically important components in soil processes such as nutrient cycling, organic matter decomposition and crop health and growth [76]. The results highlighted that the fertilization strategy strongly modified the fungal community in all studied habitats (bulk soil, root-associated soil, rhizosphere), likely due to changes in food web associations, as also reported by other studies [77,78,79]. This resulted in changes in the relative abundance, notably of the phyla Ascomycota, Glomeromycota and Mucoromycota, in all habitats. Organic fertilization (HU-org) led to the enrichment of Ascomycota and Glomeromycota, especially of the genera *Funneliformis* and *Rhizophagus* in the lettuce rhizosphere (Table 5). Zhu et al. [80] identified organic fertilization as an important factor impacting the composition and activity of mycorrhizal fungi, with the result of enhanced plant fitness.

Less is known on how high fungal pathogen abundances affect soil fungal communities. In our pot experiment, the inoculation of the pathogen led to striking shifts in the fungal community structure. Interestingly, the genus *Talaromyces* (phylum Ascomycota, order Eurotiales) predominated the fungal communities in the organic treatments (root-associated soil, rhizosphere) in the presence of *R. solani* AG1-IB (Table 6A). Marois et al. [81] suggested that organic fertilization supports the population density of *Talaromyces* in the rhizosphere, as observed also in this study. This soil-inhabiting genus, notably *T. flavus*, is known to suppress fungal pathogens such as *Verticillium dahliae* and to parasitize *R. solani* [82,83,84]. Moreover, the presence of the pathogen seems to promote *T. flavus*, which is able to produce cell wall-degrading enzymes, antifungal-acting secondary metabolites and volatile compounds that contribute to its biocontrol activity [83,85,86,87,88]. *Talaromyces* responded also in mineral-fertilized bulk soils to pathogen presence, which represents an indicator for antagonistic activity.

The genus *Rhizopus* (phylum Mucoromycota, order Mucorales) was represented by one main OTU with the closest affiliation to the saprotrophic fungus *R. arrhizus* (syn. *R. oryzae*), which dominated the soils in the present study (up to 65%) compared to our previous study (up to 3%) [38], especially in mineral fertilization in the presence of *R. solani* AG1-IB (Table 5B). Hence, Mucoromycota was one of the most dominant phyla (at least 10% relative abundance per habitat and treatment), besides Ascomycota, Basidiomycota and Mortierellomycota. The known ability of *Rhizopus* strains (e.g., *R. arrhizus*) to release 1,3–1,4-ß-glucanases and glucoamylases allows for the hydrolyzation of plant cell wall components, and thus these fungi act as decomposers [89,90]. The use of *R. solani*-infested and non-infested (control) barley kernels for inoculation may have served as a nutrient and energy source and thus explain the up to 20-fold increased relative abundance of *Rhizopus* compared to our previous study [38]. However, in contrast to the mineral treatment, the genus *Rhizopus* showed highly decreased relative abundances in the organic treatment (root-associated soil, rhizosphere) in the presence of *R. solani* AG1-IB (Table 5B). The high relative abundance of *Talaromyces* in these samples could have contributed to the decrease in *Rhizopus*. Miyake et al. [91] reported on their antagonistic activity against *Rhizopus oryzae*. However, an increased relative abundance of *Talaromyces* should then also have to be observed in the non-inoculated organic soils, but this could not be shown. Mycoparasitism of *R. solani* against *Rhizopus* was also reported earlier by Butler [92]. In contrast to the bacterial community, organic fertilization increased the alpha diversity of the fungal community, particularly in the root-associated soil, and this may have increased the competition among fungal taxa including the inoculated pathogen, as was similarly observed for wheat [77]. Additionally, the higher alpha diversity was probably due to the enrichment of fungi involved in saprophytic processes and was in accordance with our previous study [38]. The better establishment of *Rhizopus* contributed to the decreased alpha diversity in mineral-fertilized soils.

We simulated an increased density of *R. solani* AG1-IB in soil by inoculation in the pot experiment. However, a low abundance of the pathogen was revealed by molecular tools at the end of the experiment. In contrast to the expectation, a higher abundance of *R. solani* AG1-IB was determined in the bulk soil of mineral compared to organic soils but not in the rhizosphere (Table 7). We analyzed soil and rhizosphere samples after 10 weeks of lettuce growth. At earlier sampling time points, a clearer differentiation in pathogen density between the treatments could be assumed. Furthermore, it must be considered that, under field conditions, natural infestation takes place via infected plant residues and sclerotia formation [93]. This was not possible to replicate in the pot experiment and could explain the observed low abundances of the pathogen after 10 weeks.

### 4.3. Bacterial Community Shifts in Response to Fertilization Practice but Not to Pathogen Inoculation

The bacterial community structure in bulk soil and in the rhizosphere shifted in response to the fertilization strategy, similar to the findings of Chowdhury et al. [34] and Windisch et al. [38], but not in response to the pathogen *R. solani* AG1-IB. Our results confirmed the previous observation that rhizosphere bacterial communities differ significantly from those of the bulk soil. It was expected that organic fertilization increases bacterial diversity, but this was not the case. In accordance to the findings of Chowdhury et al. [34] and Schreiter et al. [44], an enrichment of, e.g., *Devosia*, *Rhizobium*, Saccharibacteria and *Asticcacaulis* in the rhizosphere of lettuce was found. In contrast to previous results with soils from the same field trial (HUB-LTE), the significant enrichment of genera belonging to Bacillales [34] by organic and of *Pseudomonadaceae* [38] by mineral fertilization in the rhizosphere was not observed in this pot experiment. Variability among rhizosphere replicates most likely hampered the ability to discriminate bacterial genera in the present study. Nevertheless, distinct rhizosphere communities differ in their ability to interact with cultivated plants and therefore affect their performance, as observed here in terms of plant gene expression in response to pathogen challenge.

The fact that AG1-IB does not attack lettuce roots [93] but the stem base and lower leaves with soil contact seems to be the reason for the only minor changes in the soil bacterial communities in the pot experiment, which is in line with the findings of Schreiter et al. [44,94] at field scale. Correspondingly, only a few taxa with significantly changed relative abundances upon pathogen inoculation were detected. In mineral-fertilized soils, the relative abundance of the actinobacterial genus *Actinoallomurus* decreased in the presence of the pathogen. Strains of *Actinoallomurus* possess several pathways for the production of secondary metabolites with antimicrobial properties [95] and have therefore the potential to directly interact with *R. solani*. However, their decreased relative abundance may indicate the strong competitiveness of *R. solani* AG1-IB. Moreover, indirect effects of the pathogen on rhizosphere bacteria via altered plant root exudation and activation of antagonistic traits must be assumed [96,97]. Gammaproteobacteria were enriched in the presence of *R. solani* in the rhizosphere of lettuce grown in organic soil. Since many members of Gammaproteobacteria are considered to be plant-beneficial [98], we suggest that their higher relative abundance in HU-org + Rs might have contributed to the defense priming of the plants and consequently to the observed upregulated gene expression.

### 4.4. R. solani AG1-IB Induced Systemic Expression of Defense-Related Genes in Lettuce Plants Grown in Soils with Long-Term Organic Fertilization

After a cultivation time of 10 weeks in the absence of *R. solani* AG1-IB, the upregulation of genes involved in (a)biotic stress responses was detected in lettuce plants when grown in organic- compared to mineral-fertilized soils (HU-org vs. HU-min; Figure 2a), as previously also found, independently of the field site [34,38]. For instance, the jasmonic acid (JA) marker gene *PDF1.2*, which results in the production of a defensin-like protein with antimicrobial functions, the salicylic acid (SA) marker gene *PR1* [99], and the *GST6* gene involved in stress protection [100] were upregulated (Figure 2a). Possibly, this observation was due to the presence of the genus *Waitea* in the indigenous fungal community, observed in higher relative abundances in organic-fertilized soil (Supplementary Table S9). This could explain the increased defense responses of lettuce against *Rhizoctonia*-like structures compared to mineral-fertilized soil. Additionally, the significant enrichment of putative pathotrophs (e.g., *Didymella*) in organic-fertilized soils, in combination with higher gene expression levels (i.a., *PDF1.2*), was in accordance with our previous study [38]. As a second possibility, it was previously discussed that the higher expression levels of defense-related genes in lettuce from organic-fertilized soils were induced by potentially beneficial microbes (e.g., Bacillales, Gammaproteobacteria) in the rhizosphere [34,38]. Rhizosphere microorganisms are able to induce *MYB72*/*BGLU42-*dependent ISR responses [101,102]. Liu et al. [103] reported on the upregulation of the gene *MYB15*, a member of the R2R3 MYB family of transcription factors, in *Arabidopsis*, under (a)biotic stress conditions. The *BGLU42* gene encodes a β-glucosidase known to play a role in plant protection through reactive oxygen species (ROS) scavenging [104]. Although no significant differences of beneficial bacterial microorganisms depending on fertilization strategy could be determined, being in contrast to our recent findings [34,38], the impact of other taxa with similar functions cannot be excluded.

In the presence of the pathogen, increased transcription levels of several genes such as *PR1*, *LOX1*, *MYC2*, *ERF104*, *ERF6*, *GST6*, *HSP70*, *BGlu42*, *OPT3*, *RbohF* and *MYB15* were found in lettuce plants grown in organic soils compared to the plants grown in mineral soils (Figure 2b). It was hypothesized that the upregulation of defense-related genes indicates ISR or “defense priming” in the plants, which may have contributed to *Rhizoctonia* disease control in the organic treatments. However, it seems that *R. solani* AG1-IB induced the observed upregulation of genes involved in plant stress responses through a direct interaction with lettuce tissue in the organic treatments (HU-org + Rs). These genes have been shown to function in (a)biotic stress signaling [103,105] and were modified by different hormone signaling pathways involved in plant immune responses. As mentioned, *PR1* is regulated by SA, while *ERF104* and *OPT3* are regulated by ethylene (ET) signaling pathways [100,106,107]. The SA- and ET/jasmonic acid (JA)-mediated signal cascades were considered to be important for plant immune responses against pathogen attacks [23]. The ability of the plants to perceive and rapidly respond to pathogens has been regarded as critical for survival. This form of first-line defense response is known as pathogen-associated molecular pattern (PAMP)-triggered immunity (PTI) and effector-triggered immunity (ETI). The enhanced expression of the SA marker gene *PR1* in leaves in the presence of the pathogen indicated the induction of such types of defense responses. The genes *RbohF*, *GST6*, *HSP70* and *OPT3* are as well involved in the regulation of ROS [108,109] and are important chemical signals in systemic acquired resistance (SAR). Pathogen recognition by the plant triggers oxidative bursts required for further defense reactions. ROS-derived signaling interacts with the essential downstream component SA of the SAR pathway [110]. Therefore, the observed induction of several genes involved in oxidative stress, SA- and ET-mediated defense responses in lettuce shoots seems to be the result of defense reactions due to encounters with effectors of the pathogen. The enhanced defense responses to *R. solani* in organic-fertilized soil could also be a result of previous priming by microbe-associated molecular patterns (MAMPs) of beneficial rhizosphere microorganisms, as found in an earlier study [34]. Moreover, the increased relative abundance of *Talaromyces* could have also induced systemic resistance in lettuce, which then showed enhanced defense gene expression in the presence of *R. solani* AG1-IB [111].

Lettuce grown in organic-fertilized soils had 23 percent less shoot growth than plants grown in mineral-fertilized soils (Figure 1), which is in line with previous findings [34,38]. All plants were facing moderate K deficiency, but in the soil with mineral fertilization, the plant K status was significantly higher compared with organic fertilization (Supplementary Table S4). This might have of course contributed to better plant growth. The pathogen reduced lettuce growth independently of the fertilization strategy (Figure 1), as also observed in previous studies [112,113]. Based on the faster spread of *R. solani* AG1-IB in organic-fertilized soils, a better establishment of the pathogen compared to mineral fertilization was expected, which results in earlier pathogen attack and thus a stronger impact on the more susceptible young lettuce plants. Indeed, a more negative impact of *R. solani* on lettuce growth was found in the organic compared to the mineral treatment (20% vs. 16%), but was lower than expected considering the spread results. However, reduced lettuce growth was observed in organic soils in the presence of the pathogen. Plant defense responses demand energy resources, which may be the reason for the lowered lettuce growth [107] and is known as the plant “growth/defense tradeoff” [114]. No differences in root dry masses in organic-fertilized soils in the presence and absence of the pathogen may support the hypothesis of higher defense reactivity. Less reduced lettuce growth due to *R. solani* attack was observed in plants grown in soils with mineral fertilization (HU-min + Rs). Based on gene expression analyses, it can be concluded that when challenged by the pathogen, the plants grown in organic soil showed enhanced expression of several genes involved in plant stress and defense signaling pathways in comparison to the plants grown in mineral soil. It could be possible that induced defense regulation helped lettuce to survive the early and continuous confrontation with the aggressive pathogen, with a tradeoff in growth. However, an additional analysis of plant stress metabolites would be helpful to answer the question of whether organic fertilization considerably improves plant health.

## 5. Conclusions

**Figure 7:**
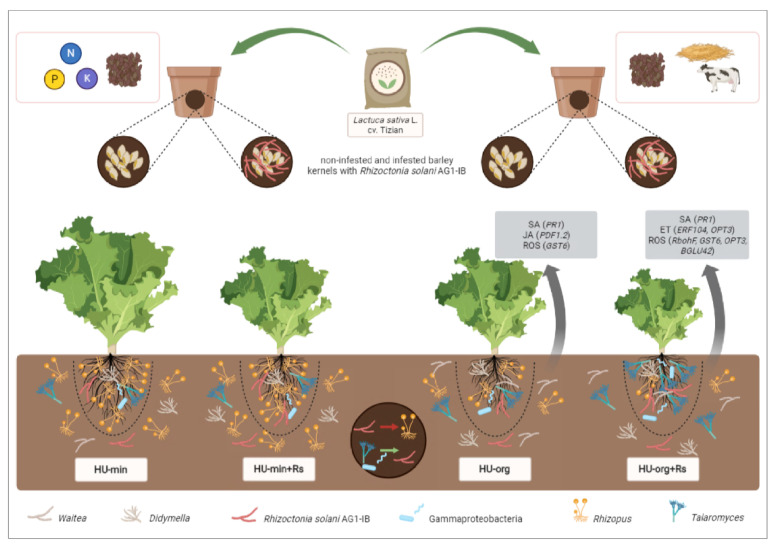
Graphical model summarizing the main results of fertilization strategy and the pathogen *Rhizoctonia solani* AG1-IB on shoot and root growth, on gene expression levels and on the most relevant microorganisms in root-associated soil (fungi, outside of the dashed lines) and in the rhizosphere (bacteria and fungi, inside of the dashed lines) of lettuce. In summary, long-term organic fertilization altered the competition of indigenous soil microorganisms and led to a better establishment of *R. solani* in organic-fertilized soils. In the absence of the pathogen, higher relative abundances of *Rhizoctonia*-like structures (*Waitea*) and putative pathotrophs (*Didymella*) in organic-fertilized soils likely resulted in increased gene expression (defense-priming). In the presence of the pathogen in organic-fertilized soils, plant-beneficial microbial taxa (*Talaromyces*, Gammaproteobacteria) were significantly enriched (green arrow) and genes that are part of defense mechanisms in a systemic response were more upregulated, resulting in reduced lettuce growth. In general, the genus *Rhizopus* was more abundant in mineral-fertilized soils and less abundant in organic-fertilized soils due to the mycoparasitism of *R. solani* (red arrow). Figure was created with BioRender.

Changes in the structure and increased diversity of the soil microbiota due to organic fertilization are postulated as possible influencing factors in the control of soil-borne phytopathogens by enabling microorganisms to enhance plant defenses and the suppression of pathogens. In contrast to mineral fertilization, organic fertilizer supported in our study the spread and activity of the *R. solani* pathogens, most probably because of their ability to efficiently use organic compounds as energy sources. In the pot experiment with lettuce/*R. solani* AG1-IB, analysis of the microbiota in the different habitats (bulk soil, root-associated soil, rhizosphere) showed that fertilization history shaped the microbial community structure (Figure 7).

In contrast to the bacterial community, organic fertilization enhanced the alpha diversity of the fungal community in root-associated soil, with consequences for the competition/interaction between the indigenous soil fungi and the artificially applied pathogen. Interestingly, the presence of *R. solani* AG1-IB shifted the fungal but not the bacterial community structure (Figure 7). In accordance with previous results, an induced physiological status (defense priming) of lettuce plants was observed in organic compared to mineral-fertilized soils. Moreover, when confronted with the pathogen *R. solani* AG1-IB, the plants grown in organic soil showed enhanced expression of genes involved in plant stress and defense signaling pathways. Interestingly, microbial taxa with putative plant-beneficial traits were enriched in the rhizosphere of lettuce grown in organic-fertilized soils in response to pathogen inoculation (e.g., *Talaromyces*, Gammaproteobacteria). Hence, it can be concluded that the upregulation of genes involved in defense pathways as a systemic response to the pathogen was probably enhanced by the priming effect of beneficial microorganisms in the rhizosphere. This was, however, compensated by retarded lettuce growth in the presence of *R. solani* AG1-IB. In summary, our results suggest that lettuce grown in soil with organic fertilization history exhibited higher fitness despite presumably better conditions for the pathogen compared to mineral fertilization. Therefore, further research is needed in order to elucidate underlying plant–microbial interactions and especially interactions between microbial populations and target pathogens under consideration of the consequences for plant health. In addition, more research regarding the effects of beneficial microorganisms enriched in response to agricultural management practices is required to support the development of sustainable plant production systems.

## Figures and Tables

**Table 1:** Disease spread of *Rhizoctonia solani* AG1-IB in lettuce (cv. Tizian) and of *R. solani* AG2-2IIIb in sugar beet (cv. Lisanna) 12 days after inoculation of the respective pathogen in soils sampled from HUB-LTE in 2015, 2016 and 2017. Small letters indicate significant differences between long-term mineral (HU-min) and organic (HU-org) fertilization using the procedure ROBUSTREG (*p* < 0.05).

Treatment	Spread of AG1-IB in Soils with Lettuce Plants [cm day^−1^]			Spread of AG2-2IIIb in Soils with Sugar Beet Plants [cm day^−1^]		
	2015	2016	2017	2015	2016	2017
HU-min	0.09 ± 0.06 b	0.25 ± 0.12 b	0.34 ± 0.09 b	0.49 ± 0.07 b	0.17 ± 0.17 b	0.59 ± 0.16 a
HU-org	0.36 ± 0.09 a	0.50 ± 0.21 a	0.41 ± 0.01 a	0.99 ± 0.13 a	0.67 ± 0.16 a	0.64 ± 0.12 a

**Table 2:** Effect of long-term mineral (HU-min) and organic (HU-org) fertilization strategy (Fertl) and the presence of the pathogen *Rhizoctonia solani* AG1-IB on 18 defense-related genes expressed in lettuce leaves (cv. Tizian). Results based on PERMANOVA analysis of ΔCt values with Bray–Curtis distances (10,000 permutations). Ev—explained variance.

Factor	Ev [%]	*p*-Value
Fertl	11.6	<0.05
*R. solani* (Rs)	5.7	n.s.
Fertl × Rs	43.7	<0.001
Residuals	38.9	

**Table 3:** Bacterial genera in bulk soils differing significantly (FDR < 0.05) in relative abundance depending on long-term organic (HU-org) and mineral (HU-min) fertilization strategy (**A**) in the absence and (**B**) in the presence of *Rhizoctonia solani* AG1-IB (+Rs). Mean ± standard deviation of taxa with >0.5% relative abundance is displayed. Bold numbers indicate significant enrichment.

(A)	Bulk Soil			
Phylum	Family	Genus	HU-org	HU-min
Acidobacteria	*Gp4* ^#^	*Gp4* ^#^	**2.3 ± 1.2**	0.4 ± 0.1
Firmicutes	*Paenibacillaceae_1*	*Fontibacillus*	**0.5 ± 0.7**	0.1 ± 0.0
Acidobacteria	*Gp1* ^#^	*Gp1* ^#^	3.0 ± 0.7	**5.4 ± 0.8**
Acidobacteria	*Occallatibacter* ^#^	*Occallatibacter* ^#^	0.0 ± 0.0	**0.5 ± 0.1**
Actinobacteria	*Solirubrobacterales* ^#^	*Solirubrobacterales* ^#^	0.5 ± 0.1	**0.8 ± 0.1**
Actinobacteria	*Thermomonosporaceae*	*Thermomonosporaceae* ^#^	0.2 ± 0.0	**0.5 ± 0.1**
Actinobacteria	*Thermomonosporaceae*	*Actinoallomurus*	0.1 ± 0.0	**0.7 ± 1.3**
Candidatus_Saccharibacteria	*Candidatus_Saccharibacteria* ^#^	*Candidatus_Saccharibacteria* ^#^	0.9 ± 0.4	**3.2 ± 1.8**
Chloroflexi	*Ktedonobacterales* ^#^	*Ktedonobacterales* ^#^	0.2 ± 0.0	**0.6 ± 0.2**
Firmicutes	*Alicyclobacillaceae*	*Tumebacillus*	0.5 ± 0.1	**1.1 ± 0.3**
Firmicutes	*Bacillaceae_1*	*Neobacillus*	1.2 ± 0.2	**2.2 ± 0.1**
Proteobacteria	*Bradyrhizobiaceae*	*Bradyrhizobium*	1.1 ± 0.3	**1.9 ± 0.6**
Proteobacteria	*Acetobacteraceae*	*Acetobacteraceae* ^#^	0.3 ± 0.1	**0.5 ± 0.1**
Proteobacteria	*Gammaproteobacteria* *_incertae_sedis*	*Acidibacter*	0.4 ± 0	**0.8 ± 0.5**
Verrucomicrobia	*Subdivision3* ^#^	*Subdivision3* ^#^	0.2 ± 0	**0.5 ± 0.2**
**(B)**	**Bulk Soil**			
**Phylum**	**Family**	**Genus**	**HU-org + Rs**	**HU-min + Rs**
Acidobacteria	*Gp4* ^#^	*Gp4* ^#^	**1.7 ± 0.6**	0.5 ± 0.1
Actinobacteria	*Micrococcaceae*	*Micrococcaceae* ^#^	**1.1 ± 1.9**	0.0 ± 0.0
Acidobacteria	*Gp1* ^#^	*Gp1* ^#^	2.4 ± 0.9	**4.7 ± 1.1**
Acidobacteria	*Gp2* ^#^	*Gp2* ^#^	0.3 ± 0.1	**0.7 ± 0.2**
Chloroflexi	*Ktedonobacterales* ^#^	*Ktedonobacterales* ^#^	0.2 ± 0.1	**0.6 ± 0.2**
Firmicutes	*Alicyclobacillaceae*	*Tumebacillus*	0.5 ± 0.2	**1.2 ± 0.3**
Firmicutes	*Bacillaceae_2*	*Bacillaceae_2* ^#^	0.2 ± 0.1	**0.7 ± 0.6**
Proteobacteria	*Proteobacteria* ^#^	*Proteobacteria* ^#^	0.3 ± 0.1	**0.5 ± 0.1**

^#^ unidentified at lower taxonomic levels.

**Table 4:** Effects of long-term mineral and organic fertilization strategy (Fertl) and the presence of the pathogen *Rhizoctonia solani* AG1-IB on fungal community composition in bulk soil (BS), root-associated soil (RA) and the rhizosphere (RH) of lettuce (cv. Tizian). PERMANOVA analysis based on Bray–Curtis distances (10,000 permutations). Ev—explained variance.

Factor	BS Ev [%]	*p*-Value	RA Ev [%]	*p*-Value	RH Ev [%]	*p*-Value
Fertl	36.9	<0.001	46.7	<0.001	30.1	<0.001
*R. solani* (Rs)	10.8	<0.05	5.8	n.s.	11.8	<0.05
Fertl × Rs	5.6	n.s.	6.4	n.s.	11.6	<0.05
Residuals	46.6		41.1		46.5	

**Table 5:** Fungal genera in bulk soil, root-associated soil and the rhizosphere of lettuce (cv. Tizian) differing significantly (FDR < 0.05) in relative abundance depending on long-term organic (HU-org) and mineral (HU-min) fertilization strategy (**A**) in the absence and (**B**) in the presence of *Rhizoctonia solani* AG1-IB (Rs). Mean ± standard deviation of taxa with >0.5% relative abundance is displayed. Bold numbers indicate significant enrichment.

(A)	Bulk Soil			Root-Associated Soil			Rhizosphere	
Genus	HU-org	HU-min	Genus	HU-org	HU-min	Genus	HU-org	HU-min
*Didymella*	**10.3 ± 2.0**	0.6 ± 0.3	*Didymella*	**8.8 ± 1.6**	0.5 ± 0.3	*Didymella*	**3.7 ± 1.7**	0.2 ± 0.1
*Ceratobasidiaceae* ^#^	**1.4 ± 2.8**	0.1 ± 0.1	*Metarhizium*	**3.5 ± 4.9**	0.6 ± 0.3	*Humicola*	**3.2 ± 5.9**	0.1 ± 0.1
*Sordariales* ^#^	**1.2 ± 0.4**	0.3 ± 0.3	*Actinomucor*	**2.4 ± 1.5**	0.0 ± 0.0	*Ilyonectria*	**1.2 ± 0.9**	0.1 ± 0.0
*Humicola*	**1.0 ± 0.2**	0.3 ± 0.1	*Powellomycetaceae* ^#^	**1.0 ± 1.2**	0.1 ± 0.1	*Arthrobotrys*	**1.0 ± 0.4**	0.1 ± 0.1
*Retroconis*	**0.9 ± 0.4**	0.3 ± 0.2	*Laetisaria*	**0.8 ± 1.0**	0.0 ± 0.0	*Plenodomus*	**0.8 ± 0.3**	0.1 ± 0.1
*Herpotrichiellaceae* ^#^	**0.8 ± 0.1**	0.2 ± 0.1	*Herpotrichiellaceae* ^#^	**0.7 ± 0.1**	0.2 ± 0.1	*Volucrispora*	**0.8 ± 0.3**	0.1 ± 0.1
*Volucrispora*	**0.5 ± 0.1**	0.1 ± 0.1	*Humicola*	**0.6 ± 0.2**	0.2 ± 0.1	*Rhizophagus*	**0.7 ± 0.8**	0.0 ± 0.0
*Laetisaria*	**0.5 ± 0.6**	0.0 ± 0.0	*Fungi* ^#^	**0.6 ± 0.3**	0.1 ± 0.0	*Actinomucor*	**0.7 ± 0.7**	0.0 ± 0.0
*Ilyonectria*	**0.5 ± 0.3**	0.0 ± 0.0	*Sordariales* ^#^	**0.5 ± 0.3**	0.1 ± 0.0	*Funneliformis*	**0.5 ± 0.3**	0.0 ± 0.0
*Rhizopus*	5.7 ± 9.8	**29.0 ± 30.5**	*Apiotrichum*	0.1 ± 0.1	**2.7 ± 4.6**	*Herpotrichiellaceae* ^#^	**0.5 ± 0.2**	0.1 ± 0.1
*Umbelopsis*	0.6 ± 0.1	**2.4 ± 1.0**	*Umbelopsis*	0.4 ± 0.1	**1.4 ± 0.7**	*Apiotrichum*	0.1 ± 0.1	**7.3 ± 14.0**
*Oculimacula*	0.2 ± 0.1	**1.2 ± 0.8**	*Paraconiothyrium*	0.0 ± 0.0	**0.7 ± 0.3**	*Talaromyces*	0.4 ± 0.5	**3.5 ± 4.4**
*Paraconiothyrium*	0.0 ± 0.0	**0.9 ± 0.2**	*Fusicolla*	0.0 ± 0.0	**0.6 ± 0.7**	*Bionectriaceae* ^#^	0.1 ± 0.1	**2.3 ± 4.5**
*Apiotrichum*	0.2 ± 0.1	**0.8 ± 0.6**	*Oculimacula*	0.2 ± 0.1	**0.5 ± 0.3**	*Saitozyma*	0.8 ± 0.5	**2.0 ± 1.5**
*Gibberella*	0.3 ± 0.1	**0.8 ± 0.9**				*Fusicolla*	0.0 ± 0.0	**1.7 ± 1.8**
*Cladophialophora*	0.2 ± 0.1	**0.5 ± 0.3**				*Umbelopsis*	0.2 ± 0.1	**1.0 ± 0.7**
						*Paraconiothyrium*	0.0 ± 0.0	**0.5 ± 0.5**
**(B)**	**Bulk Soil**			**Root-Associated Soil**			**Rhizosphere**	
**Genus**	**HU-org + Rs**	**HU-min + Rs**	**Genus**	**HU-org + Rs**	**HU-min + Rs**	**Genus**	**HU-org + Rs**	**HU-min + Rs**
*Didymella*	**8.5 ± 4.8**	0.3 ± 0.0	*Didymella*	**12.3 ± 1.3**	0.4 ± 0.1	*Talaromyces*	**51.2 ± 33.4**	3.6 ± 4.7
*Waitea*	**1.3 ± 2.5**	0.0 ± 0.0	*Talaromyces*	**11.8 ± 9.9**	0.8 ± 0.4	*Didymella*	**3.7 ± 3.7**	0.1 ± 0.0
*Rhizopus*	3.0 ± 4.7	**57.3 ± 11.6**	*Actinomucor*	**1.5 ± 2.1**	0.1 ± 0.0	*Rhizophagus*	**0.9 ± 1.6**	0.0 ± 0.0
*Umbelopsis*	0.3 ± 0.2	**1.2 ± 0.3**	*Ascobolus*	**0.9 ± 0.6**	0.1 ± 0.0	*Ilyonectria*	**0.9 ± 1.0**	0.0 ± 0.0
			*Herpotrichiellaceae* ^#^	**0.9 ± 0.4**	0.2 ± 0.0	*Rhizopus*	0.6 ± 0.3	**65.0 ± 14.9**
			*Agaricales* ^#^	**0.9 ± 0.5**	0.0 ± 0.0	*Saitozyma*	0.4 ± 0.2	**1.5 ± 1.3**
			*Tremellales* ^#^	**0.7 ± 1.1**	0.0 ± 0.0	*Umbelopsis*	0.0 ± 0.0	**0.5 ± 0.3**
			*Retroconis*	**0.5 ± 0.1**	0.1 ± 0.0			
			*Rhizopus*	4.3 ± 6.8	**54.7 ± 7.7**			
			*Umbelopsis*	0.5 ± 0.4	**1.1 ± 0.3**			
			*Sistotrema*	0.0 ± 0.0	**0.9 ± 1.6**			

^#^ unidentified at lower taxonomic levels.

**Table 6:** Fungal genera in bulk soil, root-associated soil and rhizosphere of lettuce (cv. Tizian) differing significantly (FDR < 0.05) in relative abundance depending on the absence and the presence of *Rhizoctonia solani* AG1-IB (Rs) in long-term (**A**) organic- and (**B**) mineral-fertilized soils. Mean ± standard deviation of taxa with >0.5% relative abundance is displayed. Bold numbers indicate significant enrichment.

(A)	Bulk Soil			Root-Associated Soil			Rhizosphere	
Genus	HU-org	HU-org + Rs	Genus	HU-org	HU-org + Rs	Genus	HU-org	HU-org + Rs
*Ceratobasidiaceae* ^#^	**1.4 ± 2.8**	0.0 ± 0.0	*Powellomycetaceae* ^#^	**1.0 ± 1.2**	0.2 ± 0.0	*Rhizopus*	**18.3 ± 19.2**	0.6 ± 0.3
*Volucrispora*	**0.5 ± 0.1**	0.0 ± 0.0	*Talaromyces*	0.5 ± 0.6	**11.8 ± 9.9**	*Humicola*	**3.2 ± 5.9**	0.1 ± 0.1
*Talaromyces*	0.2 ± 0.2	**24.7 ± 34.6**	*Agaricales* ^#^	0.1 ± 0.0	**0.9 ± 0.5**	*Arthrobotrys*	**1.0 ± 0.4**	0.2 ± 0.1
*Waitea*	0.0 ± 0.0	**1.3 ± 2.5**	*Tremellales* ^#^	0.0 ± 0.0	**0.7 ± 1.1**	*Volucrispora*	**0.8 ± 0.3**	0.0 ± 0.0
						*Actinomucor*	**0.7 ± 0.7**	0.0 ± 0.0
						*Gibellulopsis*	**0.6 ± 1.0**	0.0 ± 0.0
						*Talaromyces*	0.4 ± 0.5	**51.2 ± 33.4**
						*Tremellales* ^#^	0.1 ± 0.1	**4.1 ± 7.9**
						*Apiosporaceae* ^#^	0.0 ± 0.0	**0.5 ± 1.0**
**(B)**	**Bulk Soil**			**Root-Associated Soil**			**Rhizosphere**	
**Genus**	**HU-min**	**HU-min + Rs**	**Genus**	**HU-min**	**HU-min + Rs**	**Genus**	**HU-min**	**HU-min + Rs**
*Devriesia*	**0.5 ± 0.2**	0.0 ± 0.0	*Sistotrema*	0.0 ± 0.0	**0.9 ± 1.6**	*Apiotrichum*	**7.3 ± 14.0**	0.0 ± 0.0
*Talaromyces*	0.4 ± 0.4	**5.9 ± 9.7**				*Bionectriaceae* ^#^	**2.3 ± 4.5**	0.0 ± 0.0
*Cantharellales* ^#^	0.0 ± 0.0	**1.0 ± 1.9**				*Fusicolla*	**1.7 ± 1.8**	0.0 ± 0.0
						*Plenodomus*	0.1 ± 0.1	**0.7 ± 0.3**
						*Tremellales* ^#^	0.0 ± 0.0	**0.7 ± 1.0**

^#^ unidentified at lower taxonomic levels.

**Table 7:** Quantity of *Rhizoctonia solani* AG1-IB (in pg DNA per gram soil) in bulk soil (BS), root-associated soil (RA) and rhizosphere (RH) of lettuce (cv. Tizian) grown in soils with organic (HU-org) or mineral (HU-min) fertilization strategy in the absence and in the presence of *R. solani* AG1-IB (isolate 7/3/14, +Rs). Mean ± standard deviation is displayed. Different lower case characters indicate significant differences, tested separately per habitat by two-way ANOVA followed by Tukey’s test (*p* < 0.05).

Treatment	BS [pg/gram Soil]	RA [pg/gram Soil]	RH [pg/gram Soil]
HU-min	0.03 ± 0.01 ab	0.02 ± 0.01 a	0.08 ± 0.05 b
HU-min + Rs	0.09 ± 0.07 a	0.06 ± 0.09 a	1.71 ± 1.98 a
HU-org	0.04 ± 0.01 ab	0.02 ± 0.01 a	0.05 ± 0.02 b
HU-org + Rs	0.04 ± 0.04 b	0.02 ± 0.01 a	0.90 ± 1.28 a

## Data Availability

All sequence data related to this study are available in the NCBI SRA database under Accession No. PRJNA725140 (16S rRNA gene data set) or in European Nucleotide Archive (ENA) under BioProject Accession No. PRJEB53229 (ITS2 data set).

## Supplementary Materials

The following are available online at https://www.mdpi.com/article/10.3390/microorganisms10091717/s1, Figure S1: Principal coordinates analysis (PCoA, Bray–Curtis dissimilarity) based on the expression of 18 selected lettuce genes; Table S1: List of plant genes selected for expression analysis, Table S2: Primers and probes used in this study, Table S3: Overview of used Illumina barcodes and ITS2 primer combinations for each sample, Table S4: Nutritional status of lettuce (cv. Tizian), Table S5: Bacterial alpha diversity indices, Table S6: Relative abundance of the prevalent bacterial phyla, Table S7: Fungal alpha diversity indices, Table S8: Relative abundance of the prevalent fungal phyla, Table S9: Fungal genera *Thanatephorus* and *Waitea*. Reference [115] is cited in the supplementary materials.

## References

[1] Meena R.S., Kumar S., Datta R., Lal R., Vijayakumar V., Brtnicky M., Sharma M.P., Yadav G.S., Jhariya M.K., Jangir C.K. (2020). Impact of Agrochemicals on Soil Microbiota and Management: A Review. Land.

[2] Bünemann E.K., Bongiorno G., Bai Z., Creamer R.E., De Deyn G., de Goede R., Fleskens L., Geissen V., Kuyper T.W., Mäder P. (2018). Soil quality—A critical review. Soil Biol. Biochem..

[3] Sukhdev P., May P., Müller A. (2016). Fix food metrics. Nature.

[4] Martinelli F., Scalenghe R., Davino S., Panno S., Scuderi G., Ruisi P., Villa P., Stroppiana D., Boschetti M., Goulart L.R. (2015). Advanced methods of plant disease detection: A review. Agron. Sustain. Dev..

[5] Hoitink H.A.J., Boehm M.J. (1999). Biocontrol within the Context of Soil Microbial Communities: A Substrate-Dependent Phenomenon. Annu. Rev. Phytopathol..

[6] Bailey K.L., Lazarovits G. (2003). Suppressing soil-borne diseases with residue management and organic amendments. Soil Tillage Res..

[7] van Bruggen A.H.C., Semenov A.M., Finckh M.R., van Bruggen A.H.C., Tamm L. (2017). Soil Health and Soilborne Diseases in Organic Agriculture. Plant Diseases and Their Management in Organic Agriculture.

[8] van Bruggen A.H.C., Gamliel A., Finckh M.R. (2016). Plant disease management in organic farming systems. Pest Manag. Sci..

[9] Postma J., Schilder M.T., Bloem J., van Leeuwen-Haagsma W.K. (2008). Soil suppressiveness and functional diversity of the soil microflora in organic farming systems. Soil Biol. Biochem..

[10] Mendes R., Kruijt M., de Bruijn I., Dekkers E., Van Der Voort M., Schneider J.H.M., Piceno Y.M., DeSantis T.Z., Andersen G.L., Bakker P.A.H.M. (2011). Deciphering the Rhizosphere Microbiome for Disease-Suppressive Bacteria. Science.

[11] Garbeva P., Postma J., van Veen J.A., van Elsas J.D. (2006). Effect of above-ground plant species on soil microbial community structure and its impact on suppression of *Rhizoctonia solani* AG3. Environ. Microbiol..

[12] Raaijmakers J.M., Paulitz T.C., Steinberg C., Alabouvette C., Moënne-Loccoz Y. (2009). The rhizosphere: A playground and battlefield for soilborne pathogens and beneficial microorganisms. Plant Soil.

[13] Hartmann M., Frey B., Mayer J., Mäder P., Widmer F. (2015). Distinct soil microbial diversity under long-term organic and conventional farming. ISME J..

[14] Sommermann L., Geistlinger J., Wibberg D., Deubel A., Zwanzig J., Babin D., Schlüter A., Schellenberg I. (2018). Fungal community profiles in agricultural soils of a long-term field trial under different tillage, fertilization and crop rotation conditions analyzed by high-throughput ITS-amplicon sequencing. PLoS ONE.

[15] Babin D., Deubel A., Jacquiod S., Sørensen S.J., Geistlinger J., Grosch R., Smalla K. (2019). Impact of long-term agricultural management practices on soil prokaryotic communities. Soil Biol. Biochem..

[16] Allan E., Manning P., Alt F., Binkenstein J., Blaser S., Blüthgen N., Böhm S., Grassein F., Hölzel N., Klaus V.H. (2015). Land use intensification alters ecosystem multifunctionality via loss of biodiversity and changes to functional composition. Ecol. Lett..

[17] Berg G., Köberl M., Rybakova D., Müller H., Grosch R., Smalla K. (2017). Plant microbial diversity is suggested as the key to future biocontrol and health trends. FEMS Microbiol. Ecol..

[18] Hol W.H.G., De Boer W., De Hollander M., Kuramae E.E., Meisner A., Van Der Putten W.H. (2015). Context dependency and saturating effects of loss of rare soil microbes on plant productivity. Front. Plant Sci..

[19] Lapsansky E.R., Milroy A.M., Andales M.J., Vivanco J.M. (2016). Soil memory as a potential mechanism for encouraging sustainable plant health and productivity. Curr. Opin. Biotechnol..

[20] Widder S., Allen R.J., Pfeiffer T., Curtis T.P., Wiuf C., Sloan W.T., Cordero O.X., Brown S.P., Momeni B., Shou W. (2016). Challenges in microbial ecology: Building predictive understanding of community function and dynamics. ISME J..

[21] Toju H., Peay K.G., Yamamichi M., Narisawa K., Hiruma K., Naito K., Fukuda S., Ushio M., Nakaoka S., Onoda Y. (2018). Core microbiomes for sustainable agroecosystems. Nat. Plants.

[22] Francioli D., Schulz E., Lentendu G., Wubet T., Buscot F., Reitz T. (2016). Mineral vs. Organic Amendments: Microbial Community Structure, Activity and Abundance of Agriculturally Relevant Microbes Are Driven by Long-Term Fertilization Strategies. Front. Microbiol..

[23] Li H., Cai X., Gong J., Xu T., Ding G.-C., Li J. (2019). Long-Term Organic Farming Manipulated Rhizospheric Microbiome and *Bacillus* Antagonism Against Pepper Blight (*Phytophthora capsici*). Front. Microbiol..

[24] Dennert F., Imperiali N., Staub C., Schneider J., Laessle T., Zhang T., Wittwer R., Van Der Heijden M.G.A., Smits T.H.M., Schlaeppi K. (2018). Conservation tillage and organic farming induce minor variations in *Pseudomonas* abundance, their antimicrobial function and soil disease resistance. FEMS Microbiol. Ecol..

[25] van Elsas J.D., Postma J., Diaz L.F., de Bertoldi M., Bidlingmaier W., Stentiford E. (2007). Suppression of Soil-Borne Phytopathogens by Compost. Compost Science and Technology.

[26] Bonanomi G., Antignani V., Pane C., Scala F. (2007). Suppression of Soilborne Fungal Diseases with Organic Amendments. J. Plant Pathol..

[27] Yogev A., Raviv M., Hadar Y., Cohen R., Katan J. (2006). Plant waste-based composts suppressive to diseases caused by pathogenic *Fusarium oxysporum*. Eur. J. Plant Pathol..

[28] Raaijmakers J.M., Mazzola M. (2016). Soil immune responses: Soil microbiomes may be harnessed for plant health. Science.

[29] Bakker P.A.H.M., Berendsen R.L., Van Pelt J.A., Vismans G., Yu K., Li E., Van Bentum S., Poppeliers S.W.M., Sanchez Gil J.J., Zhang H. (2020). The Soil-Borne Identity and Microbiome-Assisted Agriculture: Looking Back to the Future. Mol. Plant.

[30] Semenov M.V., Krasnov G.S., Semenov V.M., van Bruggen A. (2022). Mineral and Organic Fertilizers Distinctly Affect Fungal Communities in the Crop Rhizosphere. J. Fungi.

[31] Bonanomi G., Antignani V., Capodilupo M., Scala F. (2010). Identifying the characteristics of organic soil amendments that suppress soilborne plant diseases. Soil Biol. Biochem..

[32] van Bruggen A.H.C., Sharma K., Kaku E., Karfopoulos S., Zelenev V.V., Blok W.J. (2015). Soil health indicators and Fusarium wilt suppression in organically and conventionally managed greenhouse soils. Appl. Soil Ecol..

[33] Antoniou A., Tsolakidou M.-D., Stringlis I.A., Pantelides I.S. (2017). Rhizosphere Microbiome Recruited from a Suppressive Compost Improves Plant Fitness and Increases Protection against Vascular Wilt Pathogens of Tomato. Front. Plant Sci..

[34] Chowdhury S.P., Babin D., Sandmann M., Jacquiod S., Sommermann L., Sørensen S.J., Fliessbach A., Mäder P., Geistlinger J., Smalla K. (2019). Effect of long-term organic and mineral fertilization strategies on rhizosphere microbiota assemblage and performance of lettuce. Environ. Microbiol..

[35] Beckers G.J.M., Conrath U. (2007). Priming for stress resistance: From the lab to the field. Curr. Opin. Plant Biol..

[36] Mauch-Mani B., Baccelli I., Luna E., Flors V. (2017). Defense Priming: An Adaptive Part of Induced Resistance. Annu. Rev. Plant Biol..

[37] Trost B., Prochnow A., Baumecker M., Meyer-Aurich A., Drastig K., Ellmer F. (2015). Effects of nitrogen fertilization and irrigation on N_2_O emissions from a sandy soil in Germany. Arch. Agron. Soil Sci..

[38] Windisch S., Sommermann L., Babin D., Chowdhury S.P., Grosch R., Moradtalab N., Walker F., Höglinger B., El-Hasan A., Armbruster W. (2021). Impact of Long-Term Organic and Mineral Fertilization on Rhizosphere Metabolites, Root–Microbial Interactions and Plant Health of Lettuce. Front. Microbiol..

[39] Schneider J., Schilder M., Dijst G. (1997). Characterization of *Rhizoctonia solani* AG 2 isolates causing bare patch in field grown tulips in the Netherlands. Eur. J. Plant Pathol..

[40] Verwaaijen B., Wibberg D., Nelkner J., Gordin M., Rupp O., Winkler A., Bremges A., Blom J., Grosch R., Pühler A. (2018). Assembly of the *Lactuca sativa*, L. cv. Tizian draft genome sequence reveals differences within major resistance complex 1 as compared to the cv. Salinas reference genome. J. Biotechnol..

[41] Berardini T.Z., Reiser L., Li D., Mezheritsky Y., Muller R., Strait E., Huala E. (2015). The arabidopsis information resource: Making and mining the “gold standard” annotated reference plant genome. Genesis.

[42] Livak K.J., Schmittgen T.D. (2001). Analysis of Relative Gene Expression Data Using Real-Time Quantitative PCR and the 2^−ΔΔCT^ Method. Methods.

[43] Oksanen J., Blanchet F.G., Friendly M., Kindt R., Legendre P., McGlinn D., Minchin P.R., O’Hara R.B., Simpson G.L., Solymos P. (2019). vegan: Community Ecology Package. https://cran.r-project.org/package=vegan.

[44] Schreiter S., Ding G.-C., Heuer H., Neumann G., Sandmann M., Grosch R., Kropf S., Smalla K. (2014). Effect of the soil type on the microbiome in the rhizosphere of field-grown lettuce. Front. Microbiol..

[45] Babin D., Sommermann L., Chowdhury S.P., Behr J.H., Sandmann M., Neumann G., Nesme J., Sørensen S.J., Schellenberg I., Rothballer M. (2021). Distinct rhizomicrobiota assemblages and plant performance in lettuce grown in soils with different agricultural management histories. FEMS Microbiol. Ecol..

[46] Caporaso J.G., Lauber C.L., Walters W.A., Berg-Lyons D., Lozupone C.A., Turnbaugh P.J., Fierer N., Knight R. (2011). Global patterns of 16S rRNA diversity at a depth of millions of sequences per sample. Proc. Natl. Acad. Sci. USA.

[47] Sundberg C., Al-Soud W.A., Larsson M., Alm E., Yekta S.S., Svensson B.H., Sørensen S.J., Karlsson A. (2013). 454 pyrosequencing analyses of bacterial and archaeal richness in 21 full-scale biogas digesters. FEMS Microbiol. Ecol..

[48] Yu Y., Lee C., Kim J., Hwang S. (2005). Group-specific primer and probe sets to detect methanogenic communities using quantitative real-time polymerase chain reaction. Biotechnol. Bioeng..

[49] Martin M. (2011). Cutadapt removes adapter sequences from high-throughput sequencing reads. EMBnet. J..

[50] Edgar R.C. (2013). UPARSE: Highly accurate OTU sequences from microbial amplicon reads. Nat. Methods.

[51] Schloss P.D., Westcott S.L., Ryabin T., Hall J.R., Hartmann M., Hollister E.B., Lesniewski R.A., Oakley B.B., Parks D.H., Robinson C.J. (2009). Introducing mothur: Open-Source, Platform-Independent, Community-Supported Software for Describing and Comparing Microbial Communities. Appl. Environ. Microbiol..

[52] Wang Q., Garrity G.M., Tiedje J.M., Cole J.R. (2007). Naïve Bayesian Classifier for Rapid Assignment of rRNA Sequences into the New Bacterial Taxonomy. Appl. Environ. Microbiol..

[53] Op De Beeck M., Lievens B., Busschaert P., Declerck S., Vangronsveld J., Colpaert J.V. (2014). Comparison and Validation of Some ITS Primer Pairs Useful for Fungal Metabarcoding Studies. PLoS ONE.

[54] White T.J., Bruns T., Lee S., Taylor J., Innis M.A., Gelfand D.H., Sninsky J.J., White T.J. (1990). Amplification and direct sequencing of fungal ribosomal RNA genes for phylogenetics. PCR Protocols: A Guide to Methods and Applications.

[55] Hannon G. (2010). FASTX-Toolkit. FASTQ/A Short-Reads Pre-Processing Tools. http://hannonlab.cshl.edu/fastx_toolkit/.

[56] Magoč T., Salzberg S.L. (2011). FLASH: Fast length adjustment of short reads to improve genome assemblies. Bioinformatics.

[57] Antweiler K., Schreiter S., Keilwagen J., Baldrian P., Kropf S., Smalla K., Grosch R., Heuer H. (2017). Statistical test for tolerability of effects of an antifungal biocontrol strain on fungal communities in three arable soils. Microb. Biotechnol..

[58] Kõljalg U., Larsson K.-H., Abarenkov K., Nilsson R.H., Alexander I.J., Eberhardt U., Erland S., Høiland K., Kjøller R., Larsson E. (2005). UNITE: A database providing web-based methods for the molecular identification of ectomycorrhizal fungi. New Phytol..

[59] UNITE Community (2019). UNITE General FASTA Release for Fungi: Version 18.11.2018. https://unite.ut.ee/repository.php.

[60] Wallon T., Sauvageau A., Van Der Heyden H. (2021). Detection and Quantification of *Rhizoctonia solani* and *Rhizoctonia solani* AG1-IB Causing the Bottom Rot of Lettuce in Tissues and Soils by Multiplex qPCR. Plants.

[61] R Core Team (2020). R: A language and environment for statistical computing. R Foundation for Statistical Computing. http://www.r-project.org/index.html.

[62] Kolde R. (2019). pheatmap: Pretty Heatmaps. https://cran.r-project.org/package=pheatmap.

[63] Fox J., Weisberg S. (2019). An R Companion to Applied Regression.

[64] Mangiafico S. (2020). rcompanion: Functions to Support Extension Education Program. https://cran.r-project.org/package=rcompanion.

[65] de Mendiburu F. (2020). agricolae: Statistical Procedures for Agricultural Research. https://cran.r-project.org/package=agricolae.

[66] Wickham H. (2011). The Split-Apply-Combine Strategy for Data Analysis. J. Stat. Softw..

[67] Robinson M.D., McCarthy D.J., Smyth G.K. (2010). edgeR: A Bioconductor package for differential expression analysis of digital gene expression data. Bioinformatics.

[68] McCarthy D.J., Chen Y., Smyth G.K. (2012). Differential expression analysis of multifactor RNA-Seq experiments with respect to biological variation. Nucleic Acids Res..

[69] McMurdie P.J., Holmes S. (2013). phyloseq: An R Package for Reproducible Interactive Analysis and Graphics of Microbiome Census Data. PLoS ONE.

[70] Wickham H. (2016). ggplot2: Elegant Graphics for Data Analysis.

[71] Neuwirth E. (2014). RColorBrewer: ColorBrewer Palettes. https://cran.r-project.org/package=RColorBrewer.

[72] Venables W.N., Ripley B.D. (2002). Modern Applied Statistics with S.

[73] Wang Y., Naumann U., Eddelbuettel D., Wilshire J., Warton D. (2020). mvabund: Statistical Methods for Analysing Multivariate Abundance Data. https://cran.r-project.org/package=mvabund.

[74] Grünwald N., Hu S., Van Bruggen A. (2000). Short-term Cover Crop Decomposition in Organic and Conventional Soils: Soil Microbial and Nutrient Cycling Indicator Variables Associated with Different Levels of Soil Suppressiveness to *Pythium aphanidermatum*. Eur. J. Plant Pathol..

[75] Wibberg D., Jelonek L., Rupp O., Kröber M., Goesmann A., Grosch R., Pühler A., Schlüter A. (2014). Transcriptome analysis of the phytopathogenic fungus *Rhizoctonia solani* AG1-IB 7/3/14 applying high-throughput sequencing of expressed sequence tags (ESTs). Fungal Biol..

[76] Ehrmann J., Ritz K. (2014). Plant: Soil interactions in temperate multi-cropping production systems. Plant Soil.

[77] Qiu W., Su H., Yan L., Ji K., Liu Q., Jian H. (2020). Organic Fertilization Assembles Fungal Communities of Wheat Rhizosphere Soil and Suppresses the Population Growth of *Heterodera avenae* in the Field. Front. Plant Sci..

[78] Paungfoo-Lonhienne C., Yeoh Y.K., Kasinadhuni N.R.P., Lonhienne T.G.A., Robinson N., Hugenholtz P., Ragan M.A., Schmidt S. (2015). Nitrogen fertilizer dose alters fungal communities in sugarcane soil and rhizosphere. Sci. Rep..

[79] Suleiman A.K.A., Harkes P., van den Elsen S., Holterman M., Korthals G.W., Helder J., Kuramae E.E. (2019). Organic amendment strengthens interkingdom associations in the soil and rhizosphere of barley (*Hordeum vulgare*). Sci. Total Environ..

[80] Zhu C., Ling N., Guo J., Wang M., Guo S., Shen Q. (2016). Impacts of Fertilization Regimes on Arbuscular Mycorrhizal Fungal (AMF) Community Composition Were Correlated with Organic Matter Composition in Maize Rhizosphere Soil. Front. Microbiol..

[81] Marois J.J., Fravel D.R., Papavizas G.C. (1984). Ability of *Talaromyces flavus* to occupy the rhizosphere and its interaction with *Verticillium dahliae*. Soil Biol. Biochem..

[82] Madi L., Katan T., Katan J., Henis Y. (1997). Biological Control of *Sclerotium rolfsii* and *Verticillium dahliae* by *Talaromyces flavus* Is Mediated by Different Mechanisms. Phytopathology.

[83] Kakvan N., Heydari A., Zamanizadeh H.R., Rezaee S., Naraghi L. (2013). Development of new bioformulations using *Trichoderma* and *Talaromyces* fungal antagonists for biological control of sugar beet damping-off disease. Crop Prot..

[84] Kazerooni E.A., Rethinasamy V., Al-Sadi A.M. (2019). *Talaromyces pinophilus* inhibits *Pythium* and *Rhizoctonia*-induced damping-off of cucumber. J. Plant Pathol..

[85] Kim K.K., Fravel D.R., Papavizas G.C. (1988). Identification of a Metabolite Produced by *Talaromyces flavus* as Glucose Oxidase and its Role in the Biocontrol of *Verticillium dahliae*. Phytopathology.

[86] Duo-Chuan L.I., Chen S., Jing L.U. (2005). Purification and partial characterization of two chitinases from the mycoparasitic fungus *Talaromyces flavus*. Mycopathologia.

[87] Lan D., Wu B. (2020). Chemistry and Bioactivities of Secondary Metabolites from the Genus *Talaromyces*. Chem. Biodivers..

[88] Zhai M.-M., Li J., Jiang C.-X., Shi Y.-P., Di D.-L., Crews P., Wu Q.-X. (2016). The Bioactive Secondary Metabolites from *Talaromyces* species. Nat. Prod. Bioprospect..

[89] Battaglia E., Benoit I., van den Brink J., Wiebenga A., Coutinho P.M., Henrissat B., De Vries R.P. (2011). Carbohydrate-active enzymes from the zygomycete fungus *Rhizopus oryzae*: A highly specialized approach to carbohydrate degradation depicted at genome level. BMC Genom..

[90] Celestino K.R.S., Cunha R.B., Felix C.R. (2006). Characterization of a β-glucanase produced by *Rhizopus microsporus* var. microsporus, and its potential for application in the brewing industry. BMC Biochem..

[91] Miyake T., Kato A., Tateishi H., Teraoka T., Arie T. (2012). Mode of action of *Talaromyces* sp. KNB422, a biocontrol agent against rice seedling diseases. J. Pestic. Sci..

[92] Butler E.E. (1957). *Rhizoctonia solani* as a Parasite of Fungi. Mycologia.

[93] Davis R.M., Subbarao K.V., Raid R.N. (1997). Compendium of Lettuce Diseases.

[94] Schreiter S., Babin D., Smalla K., Grosch R. (2018). Rhizosphere Competence and Biocontrol Effect of *Pseudomonas* sp. RU47 Independent from Plant Species and Soil Type at the Field Scale. Front. Microbiol..

[95] Iorio M., Tocchetti A., Cruz J.C.S., Del Gatto G., Brunati C., Maffioli S.I., Sosio M., Donadio S. (2018). Novel Polyethers from Screening *Actinoallomurus* spp.. Antibiotics.

[96] Windisch S., Bott S., Ohler M.-A., Mock H.-P., Lippmann R., Grosch R., Smalla K., Ludewig U., Neumann G. (2017). *Rhizoctonia solani* and Bacterial Inoculants Stimulate Root Exudation of Antifungal Compounds in Lettuce in a Soil-Type Specific Manner. Agronomy.

[97] Chapelle E., Mendes R., Bakker P.A.H.M., Raaijmakers J.M. (2016). Fungal invasion of the rhizosphere microbiome. ISME J..

[98] Köberl M., Dita M., Martinuz A., Staver C., Berg G. (2017). Members of Gammaproteobacteria as indicator species of healthy banana plants on *Fusarium* wilt-infested fields in Central America. Sci. Rep..

[99] Zhang X., Ménard R., Li Y., Coruzzi G.M., Heitz T., Shen W.-H., Berr A. (2020). *Arabidopsis* SDG8 Potentiates the Sustainable Transcriptional Induction of the *Pathogenesis-Related* Genes *PR1* and *PR2* During Plant Defense Response. Front. Plant Sci..

[100] Kumar S., Trivedi P.K. (2018). Glutathione S-Transferases: Role in Combating Abiotic Stresses Including Arsenic Detoxification in Plants. Front. Plant Sci..

[101] Verbon E.H., Trapet P.L., Stringlis I.A., Kruijs S., Bakker P.A.H.M., Pieterse C.M.J. (2017). Iron and Immunity. Annu. Rev. Phytopathol..

[102] Stringlis I.A., Yu K., Feussner K., de Jonge R., Van Bentum S., Van Verk M.C., Berendsen R.L., Bakker P.A.H.M., Feussner I., Pieterse C.M.J. (2018). MYB72-dependent coumarin exudation shapes root microbiome assembly to promote plant health. Proc. Natl. Acad. Sci. USA.

[103] Liu J., Osbourn A., Ma P. (2015). MYB Transcription Factors as Regulators of Phenylpropanoid Metabolism in Plants. Mol. Plant.

[104] Baba S.A., Vishwakarma R.A., Ashraf N. (2017). Functional Characterization of *Cs*BGlu12, a β-Glucosidase from *Crocus sativus*, Provides Insights into Its Role in Abiotic Stress through Accumulation of Antioxidant Flavonols. J. Biol. Chem..

[105] Otulak-Kozieł K., Kozieł E., Bujarski J.J., Frankowska-Łukawska J., Torres M.A. (2020). Respiratory Burst Oxidase Homologs RBOHD and RBOHF as Key Modulating Components of Response in Turnip Mosaic Virus—*Arabidopsis thaliana* (L.) Heyhn System. Int. J. Mol. Sci..

[106] Bethke G., Unthan T., Uhrig J.F., Pöschl Y., Gust A.A., Scheel D., Lee J. (2009). Flg22 regulates the release of an ethylene response factor substrate from MAP kinase 6 in *Arabidopsis thaliana* via ethylene signaling. Proc. Natl. Acad. Sci. USA.

[107] Garcia A., Martinez M., Diaz I., Santamaria M.E. (2021). The Price of the Induced Defense Against Pests: A Meta-Analysis. Front. Plant Sci..

[108] Dubois M., Van den Broeck L., Claeys H., Van Vlierberghe K., Matsui M., Inzé D. (2015). The ETHYLENE RESPONSE FACTORs ERF6 and ERF11 Antagonistically Regulate Mannitol-Induced Growth Inhibition in Arabidopsis. Plant Physiol..

[109] Kurt F. (2021). An Insight into Oligopeptide Transporter 3 (OPT3) Family Proteins. Protein Pept. Lett..

[110] Wendehenne D., Gao Q.-M., Kachroo A., Kachroo P. (2014). Free radical-mediated systemic immunity in plants. Curr. Opin. Plant Biol..

[111] Yamagiwa Y., Inagaki Y., Ichinose Y., Toyoda K., Hyakumachi M., Shiraishi T. (2011). *Talaromyces wortmannii* FS2 emits β-caryphyllene, which promotes plant growth and induces resistance. J. Gen. Plant Pathol..

[112] Grosch R., Dietel K., Junge H., Chowdhury S.P., Hartmann A., Borriss R., Julius-Kühn-Archiv, Julius Kühn-Institut (2012). Interaktion von *Bacillus amyloliquefaciens* FZB42 mit dem Salatfäuleerreger und der mikrobiellen Rhizosphärengemeinschaft von Salat. 58. Deutsche Pflanzenschutztagung: 10.–14. September 2012, Technische Universität Braunschweig; Kurzfassungen der Beiträge.

[113] Chowdhury S.P., Dietel K., Rändler M., Schmid M., Junge H., Borriss R., Hartmann A., Grosch R. (2013). Effects of *Bacillus amyloliquefaciens* FZB42 on Lettuce Growth and Health under Pathogen Pressure and Its Impact on the Rhizosphere Bacterial Community. PLoS ONE.

[114] Huot B., Yao J., Montgomery B.L., He S.Y. (2014). Growth–Defense Tradeoffs in Plants: A Balancing Act to Optimize Fitness. Mol. Plant.

[115] Bergmann W. (1988). Ernährungsstörungen bei Kulturpflanzen: Entstehung, visuelle u. analytische Diagnose.

