# Supplementary material for: Long-Term Fertilization Strategy Impacts Rhizoctonia solani–Microbe Interactions in Soil and Rhizosphere and Defense Responses in Lettuce
Source: Microorganisms. 2022 Aug 26;10(9):1717. doi: 10.3390/microorganisms10091717 (PMC9501836; doi:10.3390/microorganisms10091717)
Supplement: Supplementary file 1 [file microorganisms-10-01717-s001.zip › microorganisms-1828378-sm.pdf]

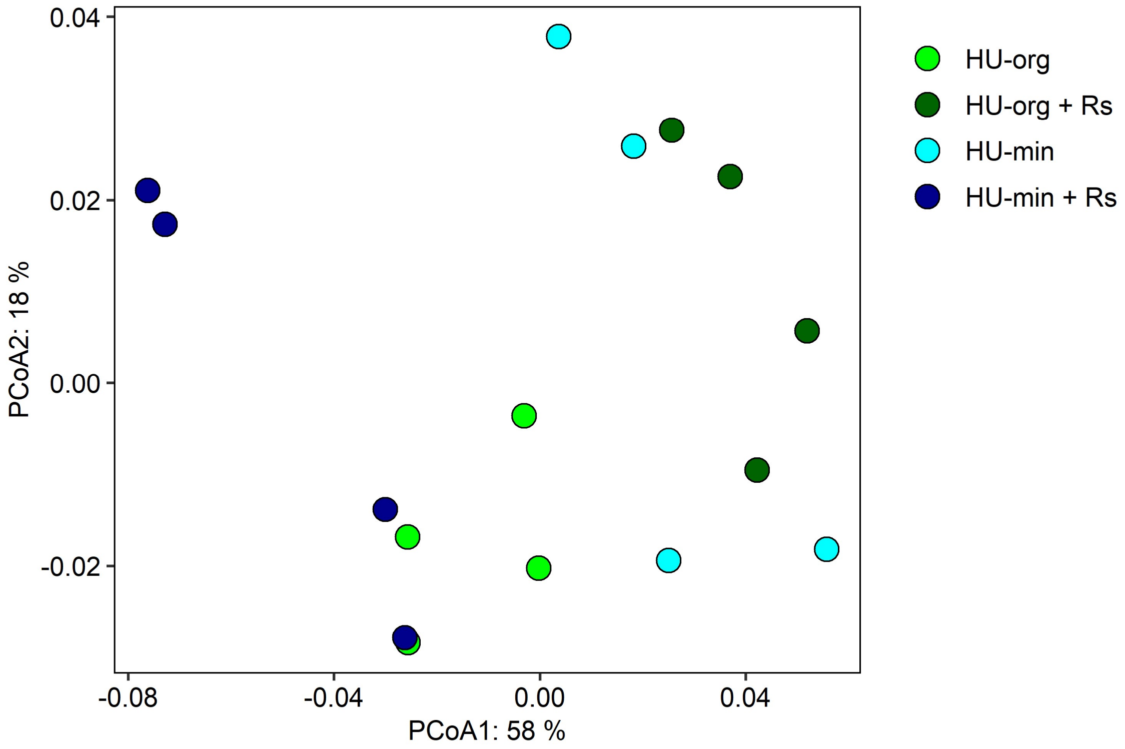

Figure S1. Principal coordinates analysis (PCoA, Bray-Curtis dissimilarity) based on the expression of 18 selected lettuce genes (ΔCt values) associated with oxidative and biotic stress signaling pathways. Lettuce (cv. Tizian) was grown in soils with long-term organic (HU-org) or mineral fertilization (HU-min), and in absence or presence of *Rhizoctonia solani* AG1-IB (HU-org+Rs, HU-min+Rs), respectively.

Table S1. List of plant genes selected for gene expression analysis with their corresponding loci, functions in *Arabidopsis thaliana* and primer sequences.

**Name of gene (Locus tag in *A. thaliana*)**	**Documented functions in *A. thaliana***	**Primer sequences (5‘ -3‘)**
OPT3 (AT4G16370)	Iron transporter involved in systemic iron, zinc and cadmium distribution within the plant.	OPTf - GGCTTGTCACCGGAATGATC<br>OPTr - TGCAAGGCGAAGAACAACAA
PR1 (AT2G14610)	Pathogenesis related protein 1, Salicylic acid (SA) dependent expression, involved in resistance against broad spectrum of pathogen.	PR1f - GAGAAGGCCGATTATGATTA<br>PR1r - ATTATTGCATTGAACCCTTG
PDF1.2 (AT5G44420)	Plant defensin factor involved in Jasmonic acid (JA)/ Ethylene (Et) dependent pathogen defense responses. Involved in Induced Systemic Resistance (ISR).	PDF1.2f - ACAAGATATGCGAGCGGAGA<br>PDF1.2r - TGACAGGCTCCATGTTTTGC
LOX1 (AT1G55020)	Lipoxigenase; upstream gene involved in the oxylipin metabolic pathway. Involved in the signaling of wounding response and JA induced defense against specific pathogens.	LOX1f - AAGAGCAGAAGCCACCCATA<br>LOX1r - GTGGAAGGAACTGCGAGAAG
WRKY70 (AT3G56400)	Transcription factor involved in both SA- and JA-mediated signal pathways. Also involved in abiotic stress signaling.	WRKY70f - GCACACACAAAACCGACCAA<br>WRKY70r - AGTTGTTGCAAGTATGGTGTCC
WRKY25 (AT2G30250)	Negative regulator of SA-mediated defense responses, elevated expression in response to oxidative stress, heat stress or wounding.	WRKY25f - TGTTCAATGAGGAAGAAGGTGG<br>WRKY25r - TCGTTTGGTGGATTGTGGTTT
MYC2 (AT1G32640)	JA-regulated transcription regulator involved in defense against bacterial pathogens/insect herbivory, flavonoid biosynthesis, and oxidative stress tolerance.	MYCf - CGGGAGCTGAATTCATTGAT<br>MYCr - CTACCGTTGACGAACGACTG
ERF104 (AT5G61600)	Et- Response Factor family transcription factor. Involved in Et- signaling and resistance to necrotrophic pathogens.	ERF104f - AGAGGAGTAAGACAACGGCC<br>ERF104r - TTTTGCTGCATCTATGGCGG
PER50 (AT4G37520)	Peroxidase; response to environmental stresses such as wounding, pathogen attack and oxidative stress.	PER50f - CTGTCAACACATGGGCTTCC<br>PER50r - TCCCACTTCGACCCGTTTTA
ERF6 (AT4G17490)	Et- Response Factor family transcription factor. Response to oxidative stress and biotic stress induced by biotrophic and necrotrophic pathogens.	ERF6f - CAAAACGTCGCGGATCTAGG<br>ERF6r - GACGCAACCTCAAGTGGAAA
ZAT10 (AT1G27730)	Zinc finger protein; transcriptional repressor involved in abiotic stress responses. Positive transcriptional regulator for salinity, heat and osmotic stress.	ZATf - TCGTGACTCCTTCCACTTCC<br>ZATr - TAGGTGGACACAAGGCTAGC

RbohF (AT1G64060)	Respiratory burst oxidase homolog F. Involved in hypersensitive reaction (HR)-related cell death and interact with intercellular ROS regulating pathogen defense responses.	RbohFf - TCATCGGCTCTAAGAAGCCC<br>RbohFr - TGCTCCAGATGACGATTACCT
GST6 (AT2G47730)	Glutathione S-transferase expressed in response to auxin, SA and hydrogen peroxide. JA-independent induction by 12-oxo-phytodienoic acid (OPDA) in plant defense.	GST6f - GCCCAAATACTTGCTCTCCG<br>GST6r - TTGGGATGACTACCGACGAG
HSP70 (AT3G12580)	Heat shock chaperone proteins induced by high light intensity, response to hydrogen peroxide, response to heat and 12-oxo-phytodienoic acid (OPDA) induced response to wounding.	HSP70f - TTCGCCTCCACCTTCTTCTT<br>HSP70r - TCACCAACGACAAGGGAAGA
LEC (AT3G15356)	Legume lectin –like protein, appears to play a role in the JA/Et response, chitin-elicited defense responses.	LEC1f - TCGTTCTCCACCTCTTTCGT<br>LEC1r - GTCCGAGATGTTGCGAAGAC
BGLU42 (AT5G36890)	The *BGLU42* gene encodes a β-glucosidase belonging to the glycosyl hydrolase family 1 (GH1) from Arabidopsis and plays a role in response to abiotic stress. Plays a role in ISR response *via* iron-mobilizing phenolics, simulating root iron-deficiency response and changes in iron-homeostasis mechanisms in the rhizosphere.	BGLUf - AGCTGAAATTCAACACGCCT<br>BGLUr - GCAAGGTGGCGAAATAGGAT
MYB15 (AT3G23250)	ABA inducible abiotic stress regulator, upregulated in cold and drought stress.	MYB15nf - AGGTGGGGTTGAAGAAAGGA<br>MYB15nr - CGTACCAGCTTTTGAAGGCA
MYB10 (AT3G12820)	JA/Ethylene-regulated, R2R3. Transcription factor involved in iron mediated response, and oxidative stress tolerance.	MYB10f - CTGGCGTTCTCTCCCAAAAC<br>MYB10r - CGACCAAAACCAATCGTGCT

Table S2. Primers used in this study for microbial community analysis.

Template	Primer/Probe	Primer/Probe sequence (5′ -3′) / Annealing-Temp. [°C]	Reference
16S rRNA gene (V3-V4, Illumina)	341F	CCTAYGGGRBGCASCAG / 56°C	Sundberg et al. 2013
	806R	GGACTACHVGGGTWTCTAAT / 56°C	Caporaso et al. 2011
ITS2 region (Illumina)	ITS86F	GTGAATCATCGAATCTTTGAA / 56°C	Op de Beeck et al. 2014
	ITS4	TCCTCCGCTTATTGATATGC / 56°C	White et al. 1990
Rhizoctonia solani (qPCR)	AG 1-IB-F3	TGGCCTTTTAACATTGGCATGT / 62°C	Wallon et al. 2021
	AG 1-IB-R	CCAACCCCAAAGGACCTTGA / 62°C	

Table S3. Overview of used Illumina barcodes and ITS2 primer combinations for each sample in bulk soil (BS), root-associated soil (RA) and the rhizosphere (RH). Data are available in the European Nucleotide Archive (ENA), (https://www.ebi.ac.uk/ena/browser/view/PRJEB53229). + Rs – in the presence of *Rhizoctonia solani* AG1-IB, HU-min – mineral fertilization, HU-org – organic fertilization.

**Run**		**Barcode + Primer (forward)**	**Barcode + Primer (reverse)**
**ERR9808751**	**ERR9808752**		
HU-org_BS1	HU-org_BS1+Rs	5'-AGGCGAAGGTGAATCATCGAATCTTTGAA-3'	5'-ATTACTCGTCCTCCGCTTATTGATATGC-3'
HU-org_BS2	HU-org_BS2+Rs		5'-TCCGGAGATCCTCCGCTTATTGATATGC-3'
HU-org_BS3	HU-org_BS3+Rs		5'-CGCTCATTTCCTCCGCTTATTGATATGC-3'
HU-org_BS4	HU-org_BS4+Rs		5'-GAGATTCCTCCTCCGCTTATTGATATGC-3'
HU-org_RA1	HU-org_RA1+Rs		5'-ATTCAGAATCCTCCGCTTATTGATATGC-3'
HU-org_RA2	HU-org_RA2+Rs		5'-GAATTCGTTCCTCCGCTTATTGATATGC-3'
HU-org_RA3	HU-org_RA3+Rs		5'-CTGAAGCTTCCTCCGCTTATTGATATGC-3'
HU-org_RA4	HU-org_RA4+Rs		5'-TAATGCGCTCCTCCGCTTATTGATATGC-3'
HU-org_RH1	HU-org_RH1+Rs		5'-CGGCTATGTCCTCCGCTTATTGATATGC-3'
HU-org_RH2	HU-org_RH2+Rs		5'-TCCGCGAATCCTCCGCTTATTGATATGC-3'
HU-org_RH3	HU-org_RH3+Rs		5'-TCTCGCGCTCCTCCGCTTATTGATATGC-3'
HU-org_RH4	HU-org_RH4+Rs		5'-AGCGATAGTCCTCCGCTTATTGATATGC-3'
HU-min_BS1	HU-min_BS1+Rs	5'-TAATCTTAGTGAATCATCGAATCTTTGAA-3'	5'-ATTACTCGTCCTCCGCTTATTGATATGC-3'
HU-min_BS2	HU-min_BS2+Rs		5'-TCCGGAGATCCTCCGCTTATTGATATGC-3'
HU-min_BS3	HU-min_BS3+Rs		5'-CGCTCATTTCCTCCGCTTATTGATATGC-3'
HU-min_BS4	HU-min_BS4+Rs		5'-GAGATTCCTCCTCCGCTTATTGATATGC-3'
HU-min_RA1	HU-min_RA1+Rs		5'-ATTCAGAATCCTCCGCTTATTGATATGC-3'
HU-min_RA2	HU-min_RA2+Rs		5'-GAATTCGTTCCTCCGCTTATTGATATGC-3'
HU-min_RA3	HU-min_RA3+Rs		5'-CTGAAGCTTCCTCCGCTTATTGATATGC-3'
HU-min_RA4	HU-min_RA4+Rs		5'-TAATGCGCTCCTCCGCTTATTGATATGC-3'
HU-min_RH1	HU-min_RH1+Rs		5'-CGGCTATGTCCTCCGCTTATTGATATGC-3'
HU-min_RH2	HU-min_RH2+Rs		5'-TCCGCGAATCCTCCGCTTATTGATATGC-3'
HU-min_RH3	HU-min_RH3+Rs		5'-TCTCGCGCTCCTCCGCTTATTGATATGC-3'
HU-min_RH4	HU-min_RH4+Rs		5'-AGCGATAGTCCTCCGCTTATTGATATGC-3'

Table S4. Nutritional status of lettuce (cv.Tizian) grown in soil under long-term mineral (HU-min) or organic (HU-org) fertilization strategy in absence and presence of the pathogen *Rhizoctonia solani* AG1-IB (+Rs). Values represent means of four repetitions ± standard deviation. Means not sharing any letters are significantly different by the Tukey-test ($p \leq 0.05$).

**Nutrients**	**DT#**	**HU-min**	**HU-min+Rs**	**HU-org**	**HU-org+Rs**
**Macro-nutrients** [g kg^{-1} shoot DM]					
N	35	21.1 ± 0.1 a	20.6 ± 0.1 a	19.0 ± 0.1 a	23.7 ± 0.1 a
P	3.0	2.5 ± 0.9 a	2.2 ± 0.9 a	2.3 ± 0.9 a	2.7 ± 1.3 a
K	42	15.2 ± 5.9 a	14.2 ± 6.1 ab	8.4 ± 4.1 c	10.6 ± 5.9 bc
Ca	12	13.1 ± 1.2 a	12.9 ± 1.5 a	13.1 ± 1.0 a	15.1 ± 4.2 a
Mg	1.0	2.0 ± 0.6 ab	1.8 ± 0.6 b	2.6 ± 0.7 ab	2.9 ± 1.1 a
S	2.5	0.5 ± 0.3 a	0.4 ± 0.1 a	0.5 ± 0.2 a	0.5 ± 0.3 a
**Micro-nutrients** [mg kg^{-1} shoot DM]					
Cu	2.5	2.0 ± 0.6 a	2.1± 0.5 a	3.0± 2.1 a	3.4 ± 1.7 a
Fe	50	49.1 ± 29.9 a	28.3± 5.6 a	36.6± 5.8 a	36.5 ± 6.9 a
Mn	20	93.6 ± 40.6 a	88.3± 33.4 a	56.4± 16.0 a	72.3± 33.1 a
Zn	20	26.5 ± 8.3 a	24.0± 8.5 a	21.9± 12.6 a	26.7± 14.5 a

Deficiency threshold (DT) for macronutrients and micronutrients [115]

Table S5. Bacterial alpha-diversity indices in bulk soil and rhizosphere of lettuce (cv. Tizian) grown in organic *vs.* mineral fertilized soil of HUB-LTE. A) *p*-values from two-way ANOVA showing the effects of fertilization strategy and inoculation as well as their interactions on alpha-diversity indices. B) Mean ± standard deviation. Different letters indicate significant differences between treatments per index and habitat tested by two-way ANOVA – Tukey's HSD test ($p \leq 0.05$). Fertl – fertilization strategy, + Rs – in the presence of *Rhizoctonia solani* AG1-IB, HU-min – mineral fertilization, HU-org – organic fertilization.

**A)**	**Richness**		**Shannon**	
**Factor**	**Bulk soil**	**Rhizosphere**	**Bulk soil**	**Rhizosphere**
Fertl	0.124	0.891	0.693	0.890
Rs	0.307	0.648	0.184	0.173
Fertl x Rs	0.076	0.937	0.480	0.964
**B)**	**Richness**		**Shannon**	
**Treatment**	**Bulk soil**	**Rhizosphere**	**Bulk soil**	**Rhizosphere**
HU-min	2150.2 ± 67.2 a	1527.1 ± 365.5 a	6.3 ± 0.1 a	4.7 ± 1.2 a
HU-min + Rs	2192.9 ± 130.8 a	1600.0 ± 196.4 a	6.2 ± 0.5 a	5.3 ± 0.2 a
HU-org	2325.5 ± 65.2 a	1556.5 ± 256.4 a	6.4 ± 0.2 a	4.8 ± 0.9 a
HU-org + Rs	2179.0 ± 109.9 a	1607.8 ± 210.2 a	6.0 ± 0.5 a	5.4 ± 0.6 a

Table S6. Relative abundance of the prevalent bacterial phyla (or proteobacterial classes) in organic *vs.* mineral fertilized soil of HUB-LTE separated per habitat (bulk soil and rhizosphere) of lettuce (cv. Tizian). Phyla with an average relative abundance of <1% were summarized as rare. *p*-values from two-way ANOVA and mean ± standard deviation showing the effects of fertilization regime, inoculation and their interactions on phyla in A) bulk soil, B) root-associated soil and C) the rhizosphere. Different letters indicate significant differences between treatments per phylum and habitat tested by two-way ANOVA – Tukey's HSD test ($P \leq 0.05$). Data was transformed by Tukey's Ladder of Power (indicated by †) if the ANOVA assumptions failed. Fertl – fertilization strategy, + Rs – in the presence of *Rhizoctonia solani* AG1-IB, HU-min – mineral fertilization, HU-org – organic fertilization.

A) **Bulk soil**

Factor	Acidobacteria	Actinobacteria	Bacteria unclass.	Bacteroidetes	Candidatus Saccharibacteria†	Chloroflexi	Firmicutes	Gemmatimonadetes	Nitrospirae†	Verrucomicrobia	Alphaproteobacteria	Betaproteobacteria	Deltaproteobacteria	Gammaproteobacteria	rare
Fertl	0.285	0.195	0.094	0.139	<0.001***	0.092	0.372	0.268	0.043*	0.762	0.373	0.721	0.054	0.444	0.041*
Rs	0.141	0.213	0.937	0.649	0.534	0.864	0.247	0.386	0.537	0.887	0.382	0.826	0.554	0.509	0.202
Fertl x Rs	0.725	0.555	0.197	0.676	0.535	0.637	0.835	0.660	0.363	0.854	0.401	0.716	0.307	0.251	0.335
**Treatment**	**Acidobacteria**	**Actinobacteria**	**Bacteria unclass.**	**Bacteroidetes**	**Candidatus Saccharibacteria†**	**Chloroflexi**	**Firmicutes**	**Gemmatimonadetes**	**Nitrospirae†**	**Verrucomicrobia**	**Alphaproteobacteria**	**Betaproteobacteria**	**Deltaproteobacteria**	**Gammaproteobacteria**	**rare**
HU-min	16.3 ± 1.9 a	13.9 ± 0.9 a	6.8 ± 0.8 a	2.2 ± 0.5 a	3.2 ± 1.8 a	1.8 ± 0.5 a	27.3 ± 6.4 a	2.4 ± 0.6 a	1.0 ± 0.1 a	1.7 ± 0 a	13.4 ± 2.6 a	3.1 ± 0.4 a	2 ± 0.3 a	3.8 ± 0.8 a	1.1 ± 0.4 a
HU-min+Rs	14.3 ± 3.3 a	11.7 ± 2.5 a	7.4 ± 1.6 a	2.2 ± 0.5 a	2.1 ± 0.6 ab	1.9 ± 0.4 a	34.2 ± 11.8 a	2.3 ± 0.6 a	0.8 ± 0.2 a	1.7 ± 0.5 a	11.3 ± 2.3 a	3.3 ± 0.8 a	2.1 ± 0.5 a	3.6 ± 0.4 a	1.1 ± 0.2 a
HU-org	15.1 ± 4.0 a	14.7 ± 1.6 a	6.6 ± 0.7 a	2.6 ± 0.4 a	0.9 ± 0.4 b	1.5 ± 0.5 a	32.8 ± 9.0 a	2.2 ± 0.6 a	1.3 ± 0.4 a	1.7 ± 0.2 a	11.2 ± 1.4 a	3.4 ± 0.7 a	1.8 ± 0.2 a	3.2 ± 0.4 a	1.0 ± 0.2 a
HU-org+Rs	11.9 ± 3.6 a	13.9 ± 3.2 a	5.8 ± 0.6 a	2.8 ± 0.8 a	0.9 ± 0.3 b	1.4 ± 0.2 a	37.7 ± 11.0 a	1.8 ± 0.7 a	1.5 ± 0.7 a	1.8 ± 0.6 a	11.2 ± 3.2 a	3.3 ± 0.6 a	1.5 ± 0.2 a	3.7 ± 0.7 a	0.7 ± 0.1 a

B) **Rhizosphere**

Factor	Acidobacteria	Actinobacteria	Bacteria unclass.†	Bacteroidetes	Candidatus Saccharibacteria	Chloroflexi	Firmicutes	Alphaproteobacteria	Betaproteobacteria	Deltaproteobacteria†	Gammaproteobacteria	rare
Fertl	0.252	0.924	0.351	0.148	0.304	0.293	0.980	0.631	0.504	0.432	0.019*	0.952
Rs	0.798	0.026*	0.574	0.988	0.383	0.131	0.040*	0.056	0.128	0.237	0.006**	0.581
Fert x Rs	0.672	0.940	0.484	0.276	0.885	0.238	0.727	0.474	0.670	0.229	0.118	0.162
**Treatment**	**Acidobacteria**	**Actinobacteria**	**Bacteria unclass.†**	**Bacteroidetes**	**Candidatus Saccharibacteria**	**Chloroflexi**	**Firmicutes**	**Alphaproteobacteria**	**Betaproteobacteria**	**Deltaproteobacteria†**	**Gammaproteobacteria**	**rare**
HU-min	2.9 ± 1.4 a	5.4 ± 2.8 a	3.0 ± 1.5 a	5.8 ± 1.9 a	3.3 ± 3.3 a	1.0 ± 0.6 a	41.9 ± 23.1 a	21.9 ± 10.6 a	7.7 ± 3.1 a	0.6 ± 0.3 a	4.5 ± 1.5 b	2.0 ± 0.5 a
HU-min+Rs	2.8 ± 1.1 a	8.7 ± 2.4 a	3.7 ± 0.8 a	6.9 ± 1.6 a	4.4 ± 2.8 a	1.8 ± 0.5 a	17.4 ± 12.3 a	35.9 ± 7.5 a	9.8 ± 3.6 a	1.1 ± 0.6 a	5.7 ± 1.0 b	1.8 ± 0.6 a
HU-org	1.9 ± 1.1 a	5.4 ± 3.1 a	3.0 ± 0.4 a	8.4 ± 2.8 a	2.2 ± 1.0 a	1.7 ± 0.4 a	38.4 ± 22.4 a	23.1 ± 12.3 a	8.1 ± 4.5 a	1.0 ± 0.1 a	5.2 ± 0.5 b	1.6 ± 0.3 a
HU-org+Rs	2.3 ± 0.8 a	8.4 ± 1.0 a	2.9 ± 0.5 a	7.2 ± 0.9 a	3.1 ± 0.7 a	1.8 ± 0.8 a	20.5 ± 12.9 a	29.9 ± 8.0 a	11.9 ± 3.3 a	1.0 ± 0.3 a	8.7 ± 2.1 a	2.2 ± 1.0 a

*$p \leq 0.05$, **$p \leq 0.01$, ***$p \leq 0.001$

Table S7. Fungal alpha-diversity indices in bulk soil (BS), root-associated soil (RA) and rhizosphere (RH) of lettuce (cv. Tizian) grown in organic *vs.* mineral fertilized soil of HUB-LTE. A) *p*-values from two-way ANOVA showing the effects of fertilization regime (Fertl) and inoculation (Rs) as well as their interactions on alpha-diversity indices. B) Mean ± standard deviation. Different letters indicate significant differences between treatments per index and habitat tested by two-way ANOVA – Tukey's HSD test ($p \leq 0.05$). Data were transformed by Tukey's Ladder of Power (indicated by †) if the ANOVA assumptions failed. Fertl – fertilization strategy, + Rs – in the presence of *Rhizoctonia solani* AG1-IB, HU-min – mineral fertilization, HU-org – organic fertilization.

**A)**	**Richness**			**Shannon**		
**Factor**	**BS**	**RA**	**RH**	**BS†**	**RA**	**RH**
Fertl	0.102	<0.001***	0.065	0.011*	0.001**	0.173
Rs	0.287	0.936	0.281	0.011*	0.206	0.194
Fertl x Rs	0.788	0.082	0.975	0.997	0.171	0.578

**B)**	**Richness**			**Shannon**		
**Treatment**	**BS**	**RA**	**RH**	**BS†**	**RA**	**RH**
HU-min	314.0 ± 30.4 a	319.0 ± 16.3 bc	243.8 ± 41.4 a	3.1 ± 1.0 ab	3.0 ± 0.7 ab	2.1 ± 0.9 a
HU-min+Rs	291.3 ± 21.2 a	295.0 ± 23.3 c	221.0 ± 29.8 a	2.2 ± 0.4 b	2.3 ± 0.4 b	1.7 ± 0.6 a
HU-org	338.5 ± 12.8 a	351.5 ± 30.8 ab	283.0 ± 30.5 a	3.9 ± 0.2 a	3.6 ± 0.5 a	3.0 ± 0.7 a
HU-org+Rs	324.8 ± 52.5 a	373.5 ± 24.4 a	261.5 ± 51.2 a	3.1 ± 1.2 ab	3.6 ± 0.1 a	2.1 ± 1.2 a

*$p \leq 0.05$, **$p \leq 0.01$, ***$p \leq 0.001$

Table S8. Relative abundance of the prevalent fungal phyla in organic *vs.* mineral fertilized soil of HUB-LTE separated per habitat (bulk soil, root-associated soil and rhizosphere) of lettuce (cv. Tizian). Phyla with an average relative abundance of <0.1% were summarized as rare. *p*-values from two-way ANOVA as well as mean ± standard deviation showing the effects of fertilization regime, inoculation and their interactions on phyla in A) bulk soil, B) root-associated soil and C) the rhizosphere. Different letters indicate significant differences between treatments per phylum and habitat tested by two-way ANOVA – Tukey's HSD test ($p \leq 0.05$). Data was transformed by Tukey's Ladder of Power (indicated by †) if the ANOVA assumptions failed. Fertl – fertilization strategy, + Rs – in the presence of *Rhizoctonia solani* AG1-IB, HU-min – mineral fertilization, HU-org – organic fertilization.

**A)**				**Bulk soil**				
**Factor**	**Asco-mycota**	**Basidio-mycota**	**Chytridio-mycota**	**Glomero-mycota†**	**Mucuro-mycota†**	**Mortierello-mycota†**	**Olpidio-mycota**	**rare**
Fertl	0.001**	0.325	<0.001***	0.002**	<0.001***	0.004**	0.069	0.023*
Rs	0.725	0.133	0.255	0.809	0.744	0.036*	0.901	0.238
Fertl x Rs	0.089	0.665	0.269	0.901	0.116	0.961	0.002**	0.883
**Treatment**	**Asco-mycota**	**Basidio-mycota**	**Chytridio-mycota**	**Glomero-mycota†**	**Mucuro-mycota†**	**Mortierello-mycota†**	**Olpidio-mycota**	**rare**
HU-min	43.2 ± 19.9 ab	13.4 ± 5.6 a	0.1 ± 0.1 b	0 ± 0 b	31.6 ± 29.5 ab	11.4 ± 5.2 ab	0 ± 0 b	0.4 ± 0.3 a
HU-min+Rs	26.9 ± 9.3 b	8.2 ± 3.2 a	0.1 ± 0.1 b	0 ± 0 ab	58.6 ± 11.4 a	6.1 ± 2.0 b	0 ± 0 ab	0.2 ± 0.1 a
HU-org	60.1 ± 9.0 a	14.8 ± 1.8 a	0.8 ± 0.2 a	0.1 ± 0.1 a	6.8 ± 9.6 bc	16.3 ± 1.0 a	0 ± 0 a	1.0 ± 0.7 a
HU-org+Rs	71.1 ± 17.5 a	11.9 ± 7.6 a	0.6 ± 0.3 a	0.3 ± 0.4 a	3.4 ± 4.9 c	12.1 ± 7.1 ab	0 ± 0 ab	0.7 ± 0.5 a
**B)**				**Root-associated soil**				
**Factor**	**Asco-mycota**	**Basidio-mycota†**	**Chytridio-mycota†**	**Glomero-mycota†**	**Mucuro-mycota**	**Mortierello-mycota**	**Olpidio-mycota**	**rare†**
Fertl	<0.001***	0.225	0.002**	<0.001***	<0.001***	0.008**	0.719	<0.001***
Rs	0.970	0.881	0.293	0.381	0.659	0.185	0.276	0.816
Fertl x Rs	0.032*	0.436	0.308	0.100	0.075	0.789	0.810	0.946
**Treatment**	**Asco-mycota**	**Basidio-mycota†**	**Chytridio-mycota†**	**Glomero-mycota†**	**Mucuro-mycota**	**Mortierello-mycota**	**Olpidio-mycota**	**rare†**
HU-min	39.4 ± 8.5 bc	13.0 ± 9.8 a	0.3 ± 0.2 b	0.1 ± 0.1 b	37.2 ± 21.5 ab	9.7 ± 4.2 ab	0.1 ± 0.1 a	0.2 ± 0.1 b
HU-min+Rs	26.8 ± 5.8 c	9.2 ± 3.0 a	0.3 ± 0.2 b	0.1 ± 0 b	56.0 ± 7.4 a	7.3 ± 2.1 b	0.1 ± 0.1 a	0.2 ± 0 b
HU-org	53.5 ± 16.3 ab	11.7 ± 3.8 a	1.5 ± 1.1 a	0.4 ± 0.2 a	18.1 ± 19.7 b	14.0 ± 2.9 a	0.2 ± 0.1 a	0.6 ± 0.3 a
HU-org+Rs	66.5 ± 8.5 a	12.8 ± 1.6 a	0.6 ± 0.1 ab	0.7 ± 0.3 a	6.4 ± 8.7 b	12.3 ± 1.8 ab	0.1 ± 0.0 a	0.6 ± 0.1 a
**C)**				**Rhizosphere**				
**Factor**	**Asco-mycota**	**Basidio-mycota†**	**Chytridio-mycota†**	**Glomero-mycota†**	**Mucuro-mycota**	**Mortierello-mycota**	**Olpidio-mycota†**	**rare**

Fertl	<0.001***	0.625	0.012*	0.002**	<0.001***	0.854	0.976	0.963
Rs	0.468	0.809	0.039*	0.830	0.818	0.653	0.148	0.156
Fertl x Rs	0.058	0.967	0.322	0.180	0.122	0.594	0.852	0.641
**Treatment**	**Asco-mycota**	**Basidio-mycota†**	**Chytridio-mycota†**	**Glomero-mycota†**	**Mucuro-mycota**	**Mortierello-mycota**	**Olpidio-mycota†**	**rare**
HU-min	32.8 ± 11.3 b	11.3 ± 17.1 a	0.1 ± 0.1 ab	0 ± 0 b	51.6 ± 31.0 ab	3.5 ± 3.5 a	0.7 ± 0.2 a	0.1 ± 0.1 a
HU-min+Rs	24.7 ± 11.3 b	3.8 ± 2.4 a	0 ± 0 b	0.3 ± 0.5 ab	65.6 ± 14.7 a	3.6 ± 2.4 a	1.8 ± 2.3 a	0.3 ± 0.3 a
HU-org	70.5 ± 16.8 a	3.2 ± 1.7 a	0.2 ± 0.1 a	1.5 ± 1.0 a	19.3 ± 19.1 bc	4.5 ± 2.1 a	0.6 ± 0.2 a	0.1 ± 0.1 a
HU-org+Rs	87.7 ± 6.9 a	5.8 ± 7.6 a	0.1 ± 0 ab	1.7 ± 2.5 a	0.7 ± 0.3 c	3.1 ± 3.1 a	0.8 ± 0.2 a	0.2 ± 0.3 a

*$p \leq 0.05$, **$p \leq 0.01$, ***$p \leq 0.001$

Table S9. Fungal genera *Thanatephorus* and *Waitea* in bulk soil (BS), root-associated soil (RA) and rhizosphere (RH) of lettuce (cv. Tizian) were analyzed by likelihood ratio tests under negative binomial distribution and generalized linear models (edgeR, FDR < 0.05) separately per habitat depending on long-term organic *vs.* mineral fertilization practice of HUB-LTE A) in the absence and B) in the presence of *Rhizoctonia solani* AG1-IB (Rs) as well as in absence vs. presence of *R. solani* AG1-IB (Rs) C) in organic and D) in mineral fertilized soils. HU-min – mineral fertilization, HU-org – organic fertilization. Relative abundances are displayed as mean ± standard deviation. Bold numbers indicate significant enrichment.

**A)**	**Bulk Soil**		**Root-associated Soil**		**Rhizosphere**	
**Genus**	**HU-org**	**HU-min**	**HU-org**	**HU-min**	**HU-org**	**HU-min**
Thanatephorus	n.d.	n.d.	n.d.	n.d.	0.002 ± 0.001	n.d.
Waitea	0 ± 0.001	n.d.	**0.064 ± 0.119**	0 ± 0.001	0.003 ± 0.003	n.d.
**B)**	**Bulk Soil**		**Root-associated Soil**		**Rhizosphere**	
**Genus**	**HU-org+Rs**	**HU-min+Rs**	**HU-org+Rs**	**HU-min+Rs**	**HU-org+Rs**	**HU-min+Rs**
Thanatephorus	0.017 ± 0.035	0.001 ± 0.002	0 ± 0.001	n.d.	0.003 ± 0.004	n.d.
Waitea	**1.286 ± 2.538**	0.008 ± 0.015	**0.016 ± 0.008**	n.d.	**0.008 ± 0.006**	n.d.
**C)**	**Bulk Soil**		**Root-associated Soil**		**Rhizosphere**	
**Genus**	**HU-org**	**HU-org+Rs**	**HU-org**	**HU-org+Rs**	**HU-org**	**HU-org+Rs**
Thanatephorus	n.d.	0.017 ± 0.035	n.d.	0 ± 0.001	0.002 ± 0.001	0.003 ± 0.004
Waitea	0 ± 0.001	**1.286 ± 2.538**	0.064 ± 0.119	0.016 ± 0.008	0.003 ± 0.003	0.008 ± 0.006
**D)**	**Bulk Soil**		**Root-associated Soil**		**Rhizosphere**	
**Genus**	**HU-min**	**HU-min+Rs**	**HU-min**	**HU-min+Rs**	**HU-min**	**HU-min+Rs**
Thanatephorus	n.d.	0.001 ± 0.002	n.d.	n.d.	n.d.	n.d.
Waitea	n.d.	0.008 ± 0.015	0 ± 0.001	n.d.	n.d.	n.d.

n.d. no sequences in any of the four replicates were detected
